# Sex differences in own and other body perception

**DOI:** 10.1002/hbm.24388

**Published:** 2018-11-15

**Authors:** Sarah M. Burke, D. S. Adnan Majid, Amir H. Manzouri, Teena Moody, Jamie D. Feusner, Ivanka Savic

**Affiliations:** ^1^ Brain & Development Research Centre, Department of Developmental and Educational Psychology Leiden University Leiden The Netherlands; ^2^ Department of Women's and Children's Health Karolinska Institute and University Hospital Stockholm Sweden; ^3^ Department of Psychiatry and Biobehavioral Sciences University of California Los Angeles Los Angeles California; ^4^ Department of Psychology Stockholm University Stockholm Sweden; ^5^ Department of Neurology University of California Los Angeles Los Angeles California

**Keywords:** body perception, fMRI, other body, own body, sex differences

## Abstract

Own body perception, and differentiating and comparing one's body to another person's body, are common cognitive functions that have relevance for self‐identity and social interactions. In several psychiatric conditions, including anorexia nervosa, body dysmorphic disorder, gender dysphoria, and autism spectrum disorder, self and own body perception, as well as aspects of social communication are disturbed. Despite most of these conditions having skewed prevalence sex ratios, little is known about whether the neural basis of own body perception differs between the sexes. We addressed this question by investigating brain activation using functional magnetic resonance imaging during a Body Perception task in 15 male and 15 female healthy participants. Participants viewed their own body, bodies of same‐sex, or opposite‐sex other people, and rated the degree that they appeared like themselves. We found that men and women did not differ in the pattern of brain activation during own body perception compared to a scrambled control image. However, when viewing images of other bodies of same‐sex or opposite‐sex, men showed significantly stronger activations in attention‐related and reward‐related brain regions, whereas women engaged stronger activations in striatal, medial‐prefrontal, and insular cortices, when viewing the own body compared to other images of the opposite sex. It is possible that other body images, particularly of the opposite sex, may be of greater salience for men, whereas images of own bodies may be more salient for women. These observations provide tentative neurobiological correlates to why women may be more vulnerable than men to conditions involving own body perception.

## 1 INTRODUCTION

The neurobiology of identity and self‐concept is currently a hot topic among neuroscientists, and emerging data suggest that it is mediated by specific cerebral networks. One fundamental facet of identity is gender. While certainly influenced by cultural and other environmental factors, gender identity is, nevertheless, foremost shaped by the perception of one's own body and its sex characteristics. Yet, we know very little about how our brain processes identification of self in the context of the sex of one's body. How does our brain distinguish own body from other bodies? Are there specific neural networks for processing recognition of the sex of the body? Are there sex differences in how cerebral networks process recognition of the physical sex in relation to self?

Self‐other distinction, crucial for human social interaction, relies mainly on the visual perception of the own and another person's body (Longo, Azañón, & Haggard, 2010). This process can be viewed as a composition of three components: (1) those involving sensory perception of own body, (2) the specific perception of body ownership, and (3) the integration of own body into the concept of self. Neural regions, within more extended networks, specialized in visual body perception include the fusiform body area (FBA) and extrastriate body area (EBA), which are specialized in human body and body parts perception (Downing & Peelen, 2016; Downing, Jiang, Shuman, & Kanwisher, 2001; Peelen & Downing, 2005; Schwarzlose, Baker, & Kanwisher, 2005). The EBA and FBA, especially on the right side, were found to be involved in *own* body representation (Peelen & Downing, 2007) and to show stronger responses after viewing pictures of one's own body compared to that of a same‐sex other (Vocks et al., 2010). These brain regions thus provide important self‐other information at a perceptual level of representation. Perception of body ownership primarily requires intact function of the temporo‐parietal junction (Limanowski & Blankenburg, 2016). Higher order social cognition (e.g., mentalizing), self‐other distinction, and (own) body representation requires recruitment of cortical midline structures: the medial prefrontal cortex (mPFC), anterior and posterior cingulate cortex, and precuneus (Northoff & Bermpohl, 2004).

More specifically, the ventral and rostral medial prefrontal cortices (mPFC) have been shown to be involved in self‐relative to other‐evaluations and in affective processing of self‐relevant information. (Amodio & Frith, 2006; Denny, Kober, Wager, & Ochsner, 2012; Murray, Schaer, & Debbané, 2012; van der Meer, Costafreda, Aleman, & David, 2010). In contrast, the dorsal mPFC was suggested to be involved in the evaluation and decision‐making process of whether a certain stimulus is applicable to the self or to another person, and was associated with judgments about dissimilar others (D'Argembeau, 2013; D'Argembeau et al., 2007; Denny et al., 2012; Mitchell, Macrae, & Banaji, 2006; Murray et al., 2012; van der Meer et al., 2010). The posterior cingulate and precuneus areas have been associated with autobiographical and semantic memory retrieval about physical aspects of own body, may be responsible for integration of self‐relevant emotional information, and have been found to be important for self‐other differentiation (Northoff & Bermpohl, 2004; Ruby & Decety, 2001; van der Cruijsen, Peters, & Crone, 2017; van der Meer et al., 2010). Of particular interest are findings in the precuneus cortex because this region is tightly connected with networks processing visual and pheromonal stimuli, and sexual arousal (Berglund, Lindström, & Savic, 2006; Cavanna & Trimble, 2006; Zhang & Li, 2012). In concert with the cortical midline structures, activation in the (anterior) insula has consistently been associated with own body awareness and ownership, integration of internal affective bodily states, and with self and familiar face processing (Craig, 2009; Kircher et al., 2001; Mega, Cummings, Salloway, & Malloy, 1997; Tsakiris, 2010; Tsakiris, Hesse, Boy, Haggard, & Fink, 2007).

Distortions of one's body image, including those that might arise during body perception, and impairments in social cognition are core symptoms of several psychiatric conditions, such as anorexia nervosa, body dysmorphic disorder, autism spectrum disorders, and in a subset of individuals with schizophrenia (American Psychiatric Association, 2013; Beilharz, Castle, Grace, & Rossell, 2017; Farrell, Lee, & Shafran, 2005; Gardner & Brown, 2014; Krumm, Ferraro, & Ingvalson, 2017; Madsen, Bohon, & Feusner, 2013; Priebe & Röhricht, 2001; Röhricht & Priebe, 1996; Ropar, Greenfield, Smith, Carey, & Newport, 2018; Smeets, Smit, Panhuysen, & Ingleby, 1997). Notably, several of these conditions show skewed sex ratios. Whereas, for example, autism spectrum disorders are more common in males than females, with a sex ratio of about 3:1 (Loomes, Hull, & Mandy, 2017), eating disorders are much more prevalent in females (Hudson, Hiripi, Pope, & Kessler, 2007; Keski‐Rahkonen & Mustelin, 2016). Body dysmorphic disorder, on the other hand, has almost equal prevalence in males and females (Buhlmann et al., 2010; Koran, Abujaoude, Large, & Serpe, 2008; Rief, Buhlmann, Wilhelm, Borkenhagen, & Brähler, 2006). A direct link between gender and own body perception also represents the hallmark of gender dysphoria, a condition gaining increasing public attention. Gender dysphoria, termed “Gender Incongruence” in the latest ICD11 criteria of the World Health Organization (https://icd.who.int/dev11/f/en#/http%3a%2f%2fid.who.int%2ficd%2fentity%2f411470068), is characterized by a perceived incongruence between a person's gender identity and his/her sex assigned at birth (DSM‐5, American Psychiatric Association, 2013). This is possibly due to a disturbed own body perception with respect to gender identity (Burke, Manzouri, Dhejne, et al., 2018; Burke, Manzouri, & Savic, 2017; Feusner, Dervisic, et al., 2016; Feusner, Lidström, et al., 2017; Manzouri, Kosidou, & Savic, 2017). Gender dysphoria has traditionally been regarded to have a male (sex assigned at birth) predominance, although this has been questioned more recently (Steensma, Cohen‐Kettenis, & Zucker, 2018; Zucker, 2017).

Whether and how own body perception differs between men and women is not known, although it has been hypothesized that women may be more sensitive to information about the own body image than men (Mitchison et al., 2017; Powell & Hendricks, 1999). One of the few studies describing sex differences in brain activations upon viewing distorted images of one's own body (appearing with different degrees of thinness or fatness) found that women showed activations in the amygdala and prefrontal areas, suggesting more complex cognitive emotional processing, whereas men had activations in the primary and secondary visual streams, similar to object and spatial visual processing (Kurosaki, Shirao, Yamashita, Okamoto, & Yamawaki, 2006). Shirao et al. (2005), investigating sex differences in brain activations during perception of negative body image related words, found amygdala activations in women, but hippocampal and prefrontal brain activations in men, suggesting a more cognitive rather than emotional processing of body image stimuli in men.

Despite vivid discussions about the representation of one's own body image in the brain (Guterstam & Ehrsson, 2012; Schauder, Mash, Bryant, & Cascio, 2015; S Vocks et al., 2011; Wiebking et al., 2010), surprisingly little is known about the neural representation of sex or gender, thus how our brain processes perception of the sex of others' bodies in relation to self, and whether this process differs between men and women (Pavlova, 2017). This issue is of special interest considering that the visual system is central for social communication, for example, for sexual attraction and partner selection. In line with this, sex differences in brain activations during body motion processing have been reported, with females showing increased activations in regions known to be involved in social cognition (Anderson et al., 2013; Pavlova, Sokolov, & Bidet‐Ildei, 2015). In addition, perception of one's own in relation to another body's sex may contribute to self‐referential processes, for example, when comparing oneself to others of the same sex (“appearance competition”) (Jackson, 1992). However, to the best of our knowledge, no study to date has investigated the neural correlates of gender identity, and sex differences in the perception of another person's body in the context of self.

We therefore developed a body perception task paradigm (Feusner, Dervisic, et al., 2016; Feusner, Lidström, et al., 2017) in which male and female participants viewed photographs of their own body, same‐sex other bodies, opposite‐sex other bodies, and sets of bodies that were morphed in increments between own body and same‐sex and opposite‐sex other bodies. For each image, the participant rated the degree that the body appeared like them: “To what degree is this picture you?” Based on previous reports, we expected to find sex differences in brain activation during *own* body perception, such that women would show stronger activations in limbic brain regions (Kurosaki et al., 2006; Shirao et al., 2005). Furthermore, we expected that both men and women during own body perception and during the perception of bodies similar to their own (i.e., same‐sex other bodies) would recruit brain areas suggested to be involved in self‐referential processing and bodily self‐consciousness (Craig, 2009; Ionta, Martuzzi, Salomon, & Blanke, 2014; Northoff, 2013; Northoff et al., 2006), such as the ventral mPFC and insula, in addition to regions involved in body perception in general (EBA and FBA). During perception of opposite‐sex bodies we predicted to find “other”‐related activations such as in the dorsal mPFC, precuneus, and TPJ (D'Argembeau et al., 2007; Eddy, 2016; Van Overwalle, 2009). Our paradigm allowed us to additionally test the novel question of whether brain activation patterns differ depending on the sex of the viewed body, independently of how that body was identified in relation to self, for instance, when the viewed body was of the opposite or same sex as the perceiver's but was in both events labeled as “not me.”

## 2 MATERIALS AND METHODS

### 2.1 Participants

We enrolled 30 healthy participants (15 males, 15 females, mean age 26 ± 3.5 years) who performed the body perception task while we acquired functional magnetic resonance imaging (fMRI) data to measure brain activity. Participants were recruited via flyers and advertisements around the campus of The Karolinska Institute. Participants had no self‐reported neurological or psychiatric disorders and were not taking any psychotropic medications. The study was approved by the ethical committee of The Karolinska Institute (application number Dnr 2011/281–31/4) and each participant provided signed informed consent before entering the study.

### 2.2 Body perception task

**Figure 1:**
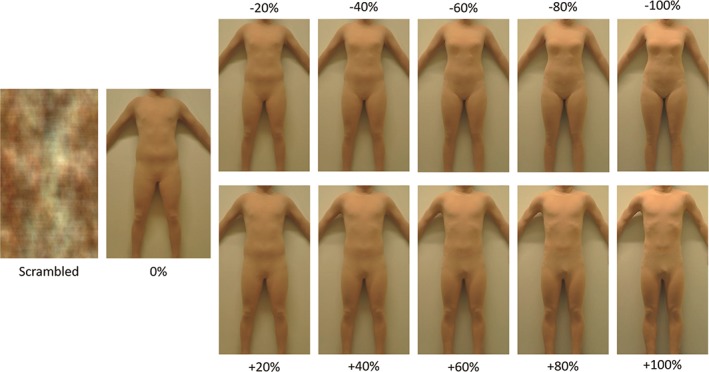
Examples of a scrambled image and a male's body images morphed, from left to right, to 20%, 40%, 60%, 80%, and 100% to the same (denoted by positive morph degrees) and the opposite (denoted by negative morph degrees) sex. Note that “100%” photographs were unaltered images of another person [Color figure can be viewed at http://wileyonlinelibrary.com]

Participants were photographed from the front with a Nikon D90, 18–105 mm f/3.5–5.6 G ED VR camera, fixed on a tripod. Lightning, contrast, and luminance were identical during each photo session. Each participant wore a skin‐colored, skin‐tight, full body unitard, and was positioned against a wall in an identical manner. The purpose of using a full‐body unitard was to best approximate the view of one's own and other bodies in the nude while avoiding the discomfort of being photographed undressed. In addition, it eliminated any differences in skin tone that would have otherwise occurred from morphing images of participants' bodies to others' bodies. Hands, feet, and head in the photos were cropped, and the photos were then morphed with photos of five other male and five other female bodies acquired in an identical manner using FantaMorph Software, version 5.0 (Abrosofthttp://www.fantamorph.com/). Each participant's picture was morphed separately with pictures from five different female and five different male participant morph targets to degrees of 20%, 40%, 60%, 80%, and 100%, respectively (producing a total of 50 different morphed images). The “100%” images were simply unaltered photos of another person. We also included the unmorphed (0% morphed) picture of each participant (Figure 1).

The total number of morph conditions was thus 11: the unmorphed 0% and images morphed 20%, 40%, 60%, 80%, and 100% to the same sex and 20%, 40%, 60%, 80%, and 100% to the opposite sex. Images were also presented over two different presentation durations: short (0.5 s) and long (2 s) durations). We present results from trials of the long 2 s duration in the main text and results from trials of the short 0.5 s duration, as well as comparisons of the two presentation durations in the supplement.

In each experiment, 15 repetitions were presented per morph percentage and for each of the two presentation durations, totaling 330 (15 × 11 × 2) experimental trials. Experimental trials were intermixed with 30 (15 for each of the short and long presentation durations) “scrambled” control images, created by phase scrambling an unmorphed body image using a Fourier phase randomization procedure (Näsänen, 1999). Here, an image's phase spectrum is replaced with random values, keeping the amplitude spectrum of the image unaltered. Global low‐level properties (i.e., luminance, contrast, color distribution, and spatial frequency spectrum) of the original image are preserved while the shape information of the image is entirely degraded. Scrambled images were also shown at two different presentation durations, 2 s and 0.5 s, and there were a total of 30 scrambled image trials.

Participants were instructed to respond as quickly as possible, rating the presented picture based on the degree to which it appeared like them, with the specific question “To what degree is this picture you?” Participants were instructed to press response button box keys 1 to 4, 1 corresponding to 0%–25% “me,” 2 to 25%–50% “me,” 3 to 50%–75% “me,” and 4 to 75%–100% “me.” Before starting the experiment, participants performed a practice session inside the scanner to ensure task comprehension.

Using Presentation version 18.1 for stimulus delivery, trials appeared in randomized order across 3 runs of 9.5 min each, acquiring 280 volumes per run. There was a 1 min break between runs. Each run began with an instruction screen, followed by a fixation cross for 30 s. Each trial consisted of (a) an image presentation for either 0.5 s or 2 s, followed by (b) the appearance of a response screen for 1 s with button press options, followed finally by (c) a fixation cross for a jittered inter‐trial interval of 1–11 s. We used optseq2 (http://surfer .http://nmr.mgh.harvard.edu/optseq/), a genetic algorithm, to create jittered presentation timing with the highest efficiency. The presentation of images was balanced and randomized with respect to degree of morph and presentation time.

### 2.3 Body localizer task

As an additional control condition and to localize those areas in the brain responsible for the specific processing of human bodies, participants performed the *body localizer task*. Participants viewed 16 alternating blocks (24 s duration) of images of either others' male or female clothed bodies (8 blocks) or chairs (8 blocks). A 10 s fixation screen was interspersed between every set of 4 blocks. To keep participants actively engaged in the task they were asked to press a button any time the exact same image (of either a chair or a body) would be presented twice in a row.

### 2.4 MR data acquisition

Magnetic resonance imaging data was acquired on a 3 Tesla MRI scanner (Discovery 3 T GE‐MR750, General Electric, Milwaukee, WI). Functional MRI of both the body perception and body localizer tasks was performed with a gradient echo pulse sequence using a voxel size of 3.03 × 3.03 × 3.5 mm (TE = 30 ms, TR = 2000 ms, FoV = 23 cm, 41 bottom up interleaved axial slices, 3 mm thickness, 75° flip angle) and a 32‐channel head coil. 3D T1‐weighted Spoiled Gradient Echo pulse sequence (SPGR) images were acquired with 1 mm^3^ isotropic voxel size (TE = 3.1 ms, TR = 7.9 ms, TI = 450 ms, FoV = 23 cm, 176 axial slices, 12° flip angle) using an 8‐channel coil.

### 2.5 Behavioral data analysis

Sample characteristics and behavioral data of the fMRI task were analyzed using SPSS Statistics 21 (SPSS Inc., Chicago, IL).

We calculated a *Self‐Perception Index* (Feusner, Lidström, et al., 2017) by multiplying the value of a participant's “self” rating (from 1 to 4) with the degree of morph. This degree of morph was 0 for the unmorphed image and was positive when images were morphed to the same‐sex other body (20%, 40%, 60%, 80%, 100%) and negative when images were morphed to the opposite‐sex other body (−20%, −40%, −60%, −80%, −100%). These weighted values were averaged for each participant and then divided by the number of rated images. Thus, greater positive values would indicate higher average “me” ratings for images morphed to a high degree to the same sex and greater negative values would indicate higher average “me” ratings for images morphed to a high degree to the opposite sex. Values closer to zero, on the other hand, would indicate higher average “me” ratings for images that were only slightly morphed from their own image.

Furthermore, male and female participants were compared with respect to their ratings of “self” when viewing their own bodies compared to bodies morphed to either the same‐ and opposite‐sex (80% and 100%, and −80% and −100% morph degrees, respectively) to evaluate possible sex differences in self‐perception.

### 2.6 MR data analysis

Data analysis was performed using FEAT (fMRI Expert Analysis Tool) version 5.0.8, part of FSL (FMRIB Software Libraryhttp://www.fmrib.ox.ac.uk/fsl) (Jenkinson, Beckmann, Behrens, Woolrich, & Smith, 2012). BOLD sequences were motion‐corrected (using the FMRIB linear image registration tool, MCFLIRT) and spatially smoothed (using FEAT) with a smoothing kernel of 5 mm. Portions of subject runs with notable movement greater than a maximum displacement of 1.5 mm were truncated if they occurred at the beginning or end of the run to minimize the effect of movement. An average of 59 TRs per run was truncated from 7 different runs of 6 subjects (3 female and 3 male controls) on account of movement. Functional images were registered to the participant's T1‐weighted image (using the FMRIB nonlinear image registration tool, FNIRT) after brain extraction using BET (implemented in FSL) with a fractional intensity threshold of 0.3. Images were then registered to the MNI‐152 brain for group analysis (using FNIRT). Higher‐level analysis was carried out first using Fixed Effects modeling to combine the three acquired runs per participant followed by a second higher‐level analysis using FLAME 1 (FMRIB's Local Analysis of Mixed Effects) for cross‐subject comparisons (Beckmann, Jenkinson, & Smith, 2003; Woolrich, 2008; Woolrich, Behrens, Beckmann, Jenkinson, & Smith, 2004). We thresholded *z*‐statistic group map images using a cluster‐forming threshold of *Z* > 2.3 and a corrected cluster significance threshold of *p* = .05. Cluster *p*‐values were determined using a spatial smoothness estimated in FSL. In addition, to further explore the extent of sex differences in (own) body perception observed, contrasts directly comparing activations of men and women were explored at a lower threshold of *Z* > 2.0, *p* < .05, corrected.

Our first set of questions was whether there are any sex differences in the perception of (1) one's own body, and (2) other bodies of the same or opposite sex, derived from images morphed 80% and 100% to same and opposite sex, respectively. Male and female participants were thus compared for the following contrasts: for (1) [own body (morphed 0%) – scrambled image]; for (2) [same‐sex other body (morphed 80–100%) – scrambled image], and [opposite‐sex other body (morphed 80%–100%) – scrambled image].

Our second set of questions was whether there are any sex‐differences in the processing of other bodies in contrast to one's own body, and if this would be affected by whether the other body is of same or opposite sex. We compared male and female participants, therefore, using the following contrasts: [same‐sex other body (morphed 80%–100%) – own body (morphed 0%)] and [opposite‐sex other body (morphed 80%–100%) – own body (morphed 0%)].

Finally, we sought to understand the neural correlates of cognitive self‐perception, utilizing participants' own behavioral measures of similarity to self as a parametric measure when viewing images morphed to either the same or opposite‐sex. Participants' responses to the question “To what degree is this picture you?” when viewing *any morphed* image (images morphed from 20% to 100%, excluding the unmorphed image of self) were parametrically modeled on a scale from 1 to 4 (see description of *Body Perception Task* above) and demeaned. Images morphed to the same‐sex and those morphed to the opposite‐sex were treated separately. This resulted in two continuous variables (for the same vs. opposite sex morphs, respectively) centered at 0, with higher values representing greater identification with “me.” In this way, neural processes involved in self‐perception could be separated from differences in perceiving same‐sex and opposite‐sex bodies of others.

## 3 RESULTS

**Figure 2:**
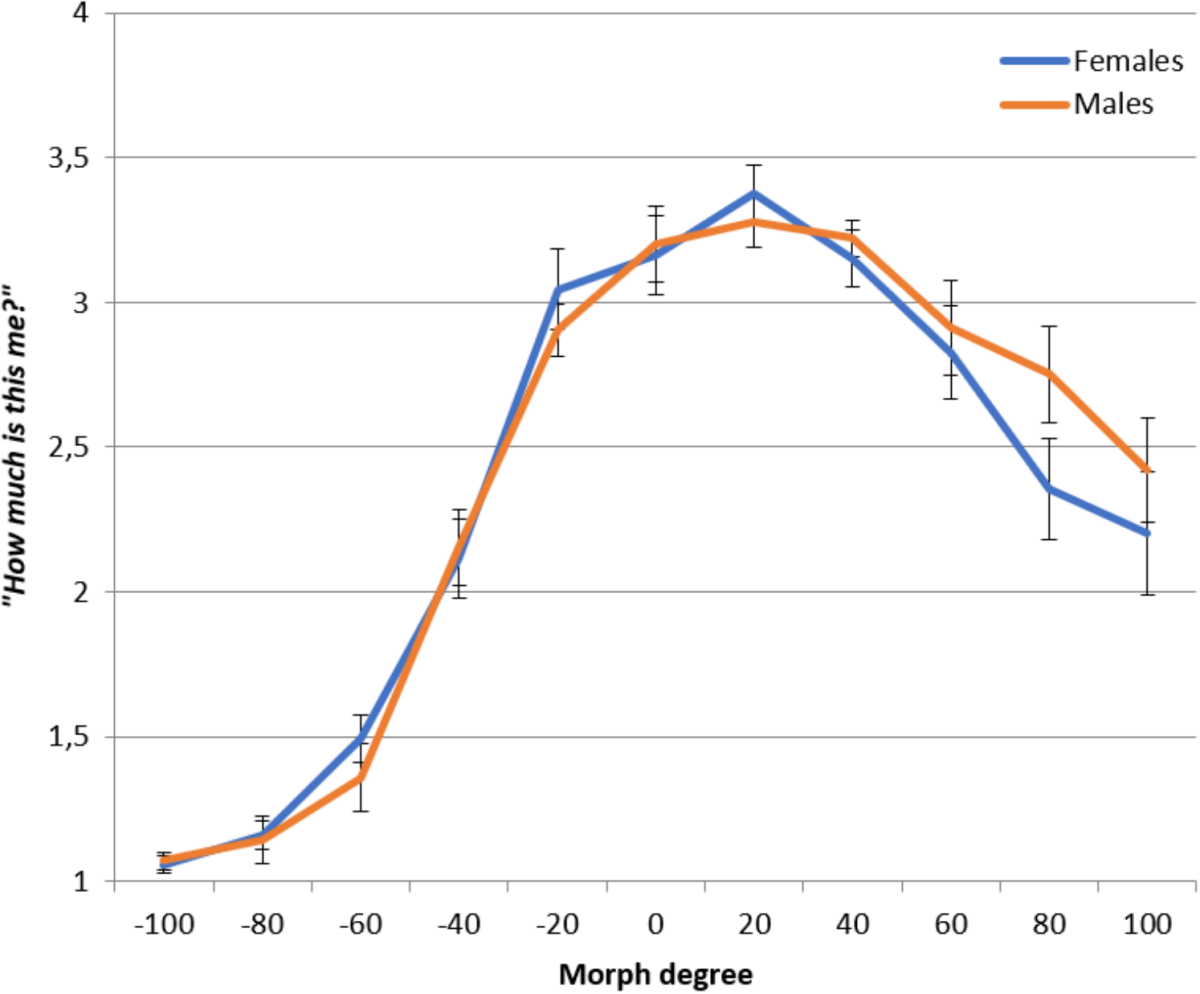
Average morph ratings for men and women for each degree of morph. Ratings ranged from 1 (0%–25% “me”) to 4 (75%–100% “me”). Positive values indicate percentage morphed to the same‐sex, whereas negative values indicate percentage morphed to the opposite‐sex. Error bars indicate standard errors of the mean. There were no significant differences between groups at any morph degree [Color figure can be viewed at http://wileyonlinelibrary.com]

Sample characteristics and self‐perception indices are presented in Table 1. Male and female participants did not differ in mean age or mean scores for handedness, and all participants identified as heterosexual. Self‐perception indices were positive for both groups, indicating, as reported earlier (Feusner, Dervisic, et al., 2016), self‐identification for images morphed to the same sex. Results from trials of the long 2 s duration are presented below, and the short 0.5 s duration results can be found in the supplement. Males' and females' ratings of self‐perception did not differ significantly at any morph degree (Figure 2).

**Table 1:** Sample characteristics and self‐perception indices

	Women		Men			
	Mean	*SD*	Mean	*SD*	T (df)	*p* value
Age	25.3	3.7	26.4	3.4	0.9 (28)	.390
Years of education	15.7	2.6	15.3	1.7	0.4 (27)	.681
Sexual orientation	0.5	0.6	0.4	0.6	0.3 (26)	.768
Handedness	62.6	73.2	78.4	48.2	0.7 (26)	.507
SP index	33.8	20.0	40.7	19.5	1.0 (28)	.343

SP = self‐perception: higher positive values, for example, indicate greater average “me” ratings for images morphed to a high degree to the same sex; sexual orientation was assessed using the self‐report Kinsey scale (Kinsey, Pomeroy, & Martin, 1948). Scores range from 0 = “exclusively heterosexual” to 6 = “exclusively homosexual” in relation to one's birth‐assigned sex; Handedness was assessed according to Oldfield (1971): scores could range from −100 (exclusively left‐handed) to +100 (exclusively right‐handed).

Despite the groups being of equivalent age, we reprocessed the analyses for all contrasts of the Body Perception Task, as presented below, using age as a covariate of no interest. The results were very similar as when age was not accounted for, and therefore are presented in the supplement (see Supporting Information Tables S9 and S10, please compare to Tables 2 and 3).

**Table 2:** Brain (de)activation for the contrast own body perception (0% morph condition) > scrambled image (control condition) in men and women

Group	*Z*‐cluster threshold	Region	Side	*x*	*y*	*z*	*Z* max	Cluster size^a^
Females (activation)	2.3	Lateral occipital cortex	R	38	−80	−2	5.73	19,905
		Postcentral gyrus	L	−42	−26	64	4.69	3,881
		Supramarginal gyrus	L	−46	−32	40	4.21	
		Superior parietal lobule	L	−28	−54	46	4.13	
		Lateral occipital cortex	R	26	−60	40	4.94	2,403
		Supramarginal gyrus	R	44	−36	46	4.79	
		Superior parietal lobule	R	30	−52	40	4.43	
		Paracingulate gyrus	R	6	26	42	4.46	1869
		Superior frontal gyrus	R	2	42	38	3.52	
		Precentral gyrus	R	46	8	30	5.54	975
		Insular cortex	R	42	0	6	3.48	
Males (activation)	2.3	Occipital pole	L	−32	−92	−4	5.21	14,349
		Lateral occipital cortex	L	−34	−88	4	4.9	
		Middle frontal gyrus	R	48	32	22	4.87	
		Lateral occipital cortex	R	48	−76	−8	5.71	5,864
		Inferior temporal gyrus	R	50	−56	−6	5.1	
		Paracingulate gyrus	R	4	26	46	4.56	1,030
		Superior frontal gyrus	L	−2	12	58	3.63	
Females (deactivation)	2.3	Occipital pole	L	−14	−92	14	5.19	5,009
		Lingual gyrus, temporal occipital fusiform cortex	L	−26	−56	−6	4.83	
		Lateral occipital cortex	L	−18	−88	26	4.35	
		Temporo‐parietal junction	R	56	−50	24	5.33	4,444
		Temporo‐parietal junction	L	−64	−48	2	4.68	2,849
		Posterior cingulate	R	10	−32	46	4.79	2,134
		Precuneus cortex	R	10	−36	46	4.68	
Males (deactivation)	2.3	Occipital pole	R	12	−90	16	4.66	3,876
		Occipital fusiform gyrus	R	26	−66	−8	4.5	
		Lingual gyrus, temporal occipital fusiform cortex	R	26	−56	−8	4.39	
		Temporo‐parietal junction	R	52	−48	14	4.61	3,690
		Temporo‐parietal junction	L	−60	−54	14	4.73	1,103

a Cluster size is number of voxels with voxel size of 3 × 3 × 3.5 mm; R = right; L = left.

**Table 3:** Sex‐differences in brain activation

Contrast	Group	*Z*‐cluster threshold	Region	Side	*x*	*y*	*z*	*Z* max	Cluster size^a^
Body localizer task (bodies > chairs)	M > F	2.3	Postcentral gyrus	R	36	−32	44	3.33	843
			Precentral gyrus	R	34	−26	60	3	
			Superior frontal gyrus	R	22	2	66	2.99	
			Precentral gyrus, superior frontal gyrus	L	−30	−4	68	3.54	551
			Middle frontal gyrus	L	−28	−2	58	3.23	
Same‐sex morph > scramble	M > F	2.3	Lateral occipital cortex (EBA)	L	−28	−78	22	3.54	501
		2.0	Posterior cingulate and Precuneus	M	0	−36	48	3.57	869
Same‐sex morph > own body (0% morph)	M > F	2.0	Lateral occipital cortex (EBA)	L	−42	−76	−2	3.52	1,626
			Temporal occipital fusiform cortex (FBA)	L	−40	−54	−12	3.32	
			Lateral occipital cortex (EBA)	R	50	−64	−2	3.19	797
			Occipital fusiform cortex (FBA)	R	38	−70	−10	3.01	
Opposite‐sex morph > scramble	M > F	2.3	Lateral occipital cortex (EBA)	L	−22	−82	26	3.75	1,396
			Precuneus cortex	L	−2	−38	48	4.13	1,395
			Posterior cingulate	R	4	−36	48	3.64	
			Middle temporal gyrus	L	−52	−44	6	3.51	1,020
			Frontal pole	L	−28	40	34	3.91	565
			Temporo‐parietal junction	R	42	−50	26	3.50	512
			Occipital fusiform gyrus (FBA)	R	18	−80	−10	3.21	488
		2.0	Caudate nucleus	L	−16	20	6	3.47	2042
			Caudate nucleus	R	18	24	10	3.20	
			Inferior frontal gyrus	L	−48	14	28	3.44	
Opposite‐sex morph > own body (0% morph)	M > F	2.3	Cuneal and Precuneus cortices	R	4	−78	36	3.49	1,352
			Supracalcarine cortex	R	2	−72	16	3.34	
			Intracalcarine cortex and lingual gyrus	R	4	−72	8	3.10	
		2.0	Frontal orbital cortex	L	−34	22	−12	3.41	1,235
			Caudate nucleus	L	−8	4	2	3.31	
			Nucleus Accumbens	L	−8	4	2	3.11	
			Insular cortex	L	−36	6	−10	2.82	
			Temporo‐parietal junction	R	50	−40	14	3.34	1,126
			Paracingulate	R	4	34	36	2.85	1,032
			Caudate nucleus	R	18	18	0	3.27	982
			Putamen	R	14	10	−6	3.00	
			Insular cortex	R	32	12	−12	3.92	
			Frontal pole	R	34	52	26	3.00	818
Parametric association of greater “not me” rating during opposite‐sex morph viewing	M > F	2.3	Precuneus and posterior cingulate	R	8	−50	16	3.74	7,562
			Amygdala	L	−20	−6	−14	3.66	
			Amygdala	R	18	0	−16	3.47	

a cluster size is number of voxels with voxel size of 3 × 3 × 3.5 mm; R = right; L = left; EBA = extrastriate body area; FBA = fusiform body area.

### 3.1 Body localizer task

As has been shown in previous studies, the *body localizer task* resulted in significant (*Z* > 2.3, *p* < .05, corrected) bilateral activation in areas specialized for body perception, in both males and females. These areas included bilateral lateral occipital cortices (EBA), temporal occipital fusiform gyri (FBA), precuneus, left angular gyrus, bilateral precentral gyri, and the right amygdala in males (see Supporting Information Table S1). When comparing males and females, males showed significantly (*Z* > 2.3, *p* < .05, corrected) greater activation in the bilateral motor cortex and superior frontal gyri (Table 3).

### 3.2 Own body perception

On account of an error, one male participant did not see images of his own body but rather another participant's body during the scan. This participant was therefore excluded in all analyses involving *own body*.

Contrasting perception of one's *own body* (0% morph) with the *scrambled* image baseline revealed significant (*Z* > 2.3, *p* < .05, corrected) activation in both men (N = 14) and women (N = 15) in the bilateral lateral occipital cortex, including the EBA, dorsal medial PFC, bilateral frontal operculum/anterior insula, caudate nucleus, and thalamus. There were no significant differences between groups (Supporting Information Figure S1 and Table 2). Both males and females showed right dominant deactivation in the precuneus, posterior cingulate, TPJ (bilateral, but right‐dominant middle temporal gyri, angular gyri, supramarginal gyri), right temporal pole, and fusiform gyri (Table 2 and Supporting Information Table S2).

### 3.3 Same‐sex other body perception

Contrasting perception of *other bodies of the same sex* (80–100% morph) with the *scrambled* image baseline revealed significant (*Z* > 2.3, *p* < .05, corrected) activation in both (N = 15) men and (N = 15) women in the bilateral inferior lateral occipital cortices (EBA), fusiform cortices (FBA), bilateral caudate nucleus, thalamus, bilateral anterior insula, ventrolateral PFC, and dorsal mPFC, anterior cingulate cortices, and bilateral cerebellar hemispheres (Supporting Information Figure S1). In both groups, there was deactivation of the bilateral TPJ (middle temporal gyri, angular gyri, supramarginal, gyri) (Supporting Information Table S3). When comparing males and females, males showed significantly (*Z* > 2.3, *p* < .05, corrected) greater activation in the left superior lateral occipital cortex. Using a slightly more lenient threshold of *Z* > 2.0, *p* < .05, corrected, revealed additional, stronger activation in the precuneus cortex of males. The latter effect, however, was due to greater *de*activation in this area in females during perception of same‐sex other bodies (Table 3 and Supporting Information Table S3).

**Figure 3:**
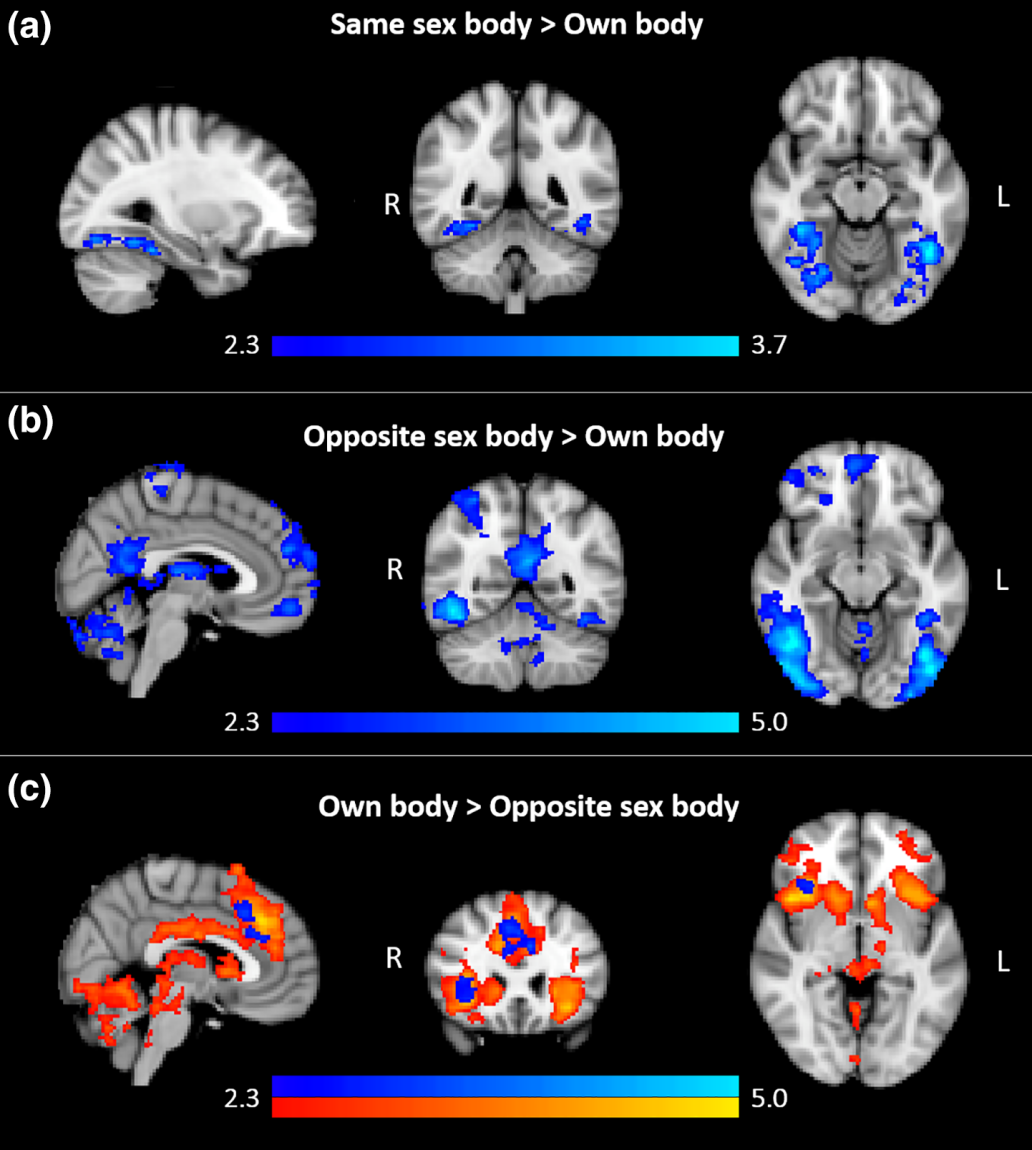
Brain activation in men (blue‐light blue color) and women (red‐yellow color) when viewing images of (a) a same sex other body and (b) an opposite sex other body, contrasted to images of the own body, respectively, and (c) when viewing images of the own body contrasted to images of an opposite sex other body; MNI coordinates of the slices shown: (a) *x* = 30, *y* = −48, *z* = −14; (b) *x* = 4, *y* = −54, *z* = −12; (c) *x* = 4, *y* = 24, *z* = −4; R = right, L = left; color bars indicate *z* value of the presented contrast

**Figure 4:**
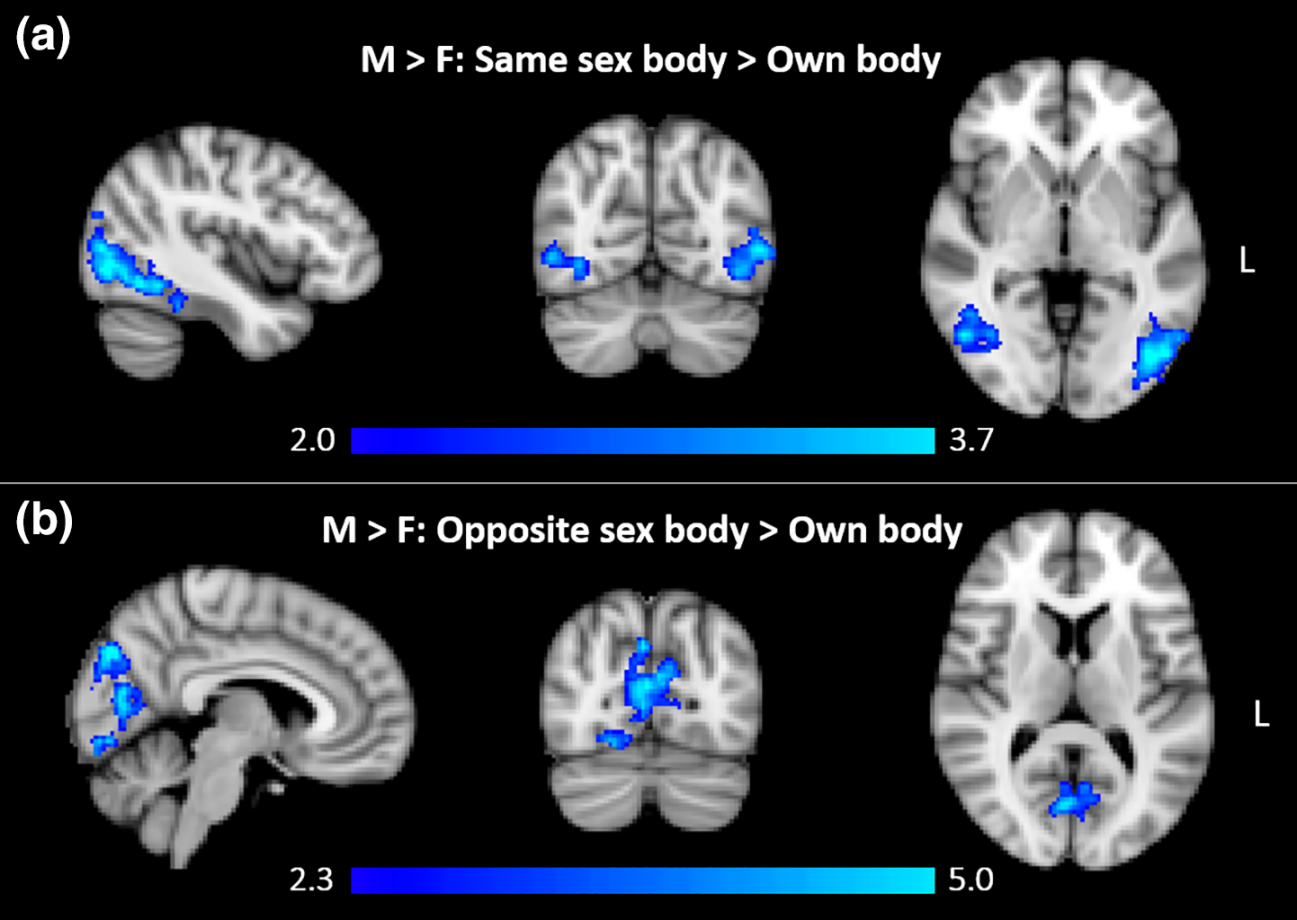
Sex differences in activation, with men (M) showing greater activation than women (F) when viewing images of (a) a same sex other body and (b) an opposite sex other body, contrasted to images of the own body, respectively; MNI coordinates of the slices shown: (a) *x* = −42, *y* = −66, *z* = −2; (b) *x* = 6, *y* = −74, *z* = 10; R = right, L = left; color bars indicate *z* value of the presented contrast

Contrasting perception of other bodies of the *same sex* (80%–100% morph) with one's *own body* (0% morph) revealed significant (*Z* > 2.3, *p* < .05, corrected) activation only in males in the bilateral temporal occipital and fusiform cortex (EBA, FBA), left precentral gyrus, and left ventrolateral PFC (Figure 3). Women showed no significant differences in activation between perception of the own body and perception of other females' body. Although there were no significant differences between females and males at the *Z* > 2.3 threshold, lowering the threshold to *Z* > 2.0 revealed that men had significantly greater activation in the bilateral FBA and bilateral lateral occipital cortex (EBA) (Table 3, Figure 4).

### 3.4 Opposite‐sex other body perception

Contrasting perception of other bodies of the *opposite sex* (80%–100% morph) with the *scrambled* control images revealed significant (*Z* > 2.3, *p* < .05, corrected) activation in both men and women in the bilateral lateral occipital cortex including the EBA and FBA, and the right dorsolateral PFC. Both groups showed significant deactivation in the angular and supramarginal gyri. Women, in addition, showed deactivations in the precuneus and left frontal pole. Direct comparison of men and women revealed significantly greater activation in men in the bilateral EBA and FBA, precuneus, left middle temporal gyrus, right‐TPJ (angular, superior temporal, and supramarginal gyri), and left frontal pole (Supporting Information Figure S1 and Table S5). Using a slightly more lenient threshold (*Z* > 2.0, *p* < .05, corrected), men showed additional stronger activations compared to women in the bilateral caudate nucleus and left inferior frontal gyrus (Table 3).

Contrasting perception of other bodies of the *opposite sex* (80%–100% morph) with one's *own body* (0% morph) revealed no significant (*Z* > 2.3, *p* < 0.05, corrected) activations in women, whereas in men there was significant activation in the (pre)cuneus cortex, bilateral TPJ (supramarginal, superior temporal, angular gyri), and right middle temporal gyrus (both anterior and posterior parts) (Figure 3). The direct group comparison revealed significantly stronger activations in men than in women in the bilateral precuneus, supra‐ and intracalcarine cortices, and lingual gyri (Figure 4). With a threshold of *Z* = 2.0, *p* < 0.05, corrected, men showed additional greater activations than women in the bilateral caudate nucleus and left accumbens, frontal pole, right‐TPJ (supramarginal, middle temporal, superior temporal, angular gyri), and the bilateral anterior insular cortices (Table 3).

By contrast, women showed pronounced deactivation (i.e., greater activation to their *own* bodies compared to opposite sex bodies) in the bilateral anterior insula, right anterior cingulate cortex, left cerebellum, left postcentral gyrus, left precuneus, and bilateral (though right‐dominant) TPJ (Figure 3c). Deactivations (activation to their *own* bodies more than to opposite sex bodies) in males were detected in the bilateral anterior cingulate gyri, right ventrolateral PFC, right‐anterior insula, and right‐superior parietal lobule (Figure 3c). Thus, greater activations in response to own body compared with opposite sex bodies were observed in both men and women, but more pronounced in women. This indicates that the sex difference pattern in regions such as the anterior insula and right TPJ was driven by greater activation to own bodies than opposite sex other bodies in the women, rather than greater activation for opposite sex other bodies in men.

### 3.5 Response‐dependent perception of images morphed to same‐sex and opposite‐sex other bodies

When viewing images morphed to the *same sex* (20%–100%), participants' ratings of greater self‐similarity (greater “me” rating) was significantly (*Z* > 2.3, *p* < 0.05, corrected) associated with activation in the left postcentral gyrus in both males and females. Participants' ratings of greater self‐similarity (greater “me” rating), when viewing images morphed to the *opposite sex* (20%–100%), was significantly (*Z* > 2.3, *p* < .05, corrected) associated with activation in the bilateral insula, anterior cingulate, and paracingulate in both males and females.

By contrast, participants' rating of less self‐similarity (greater “not me” rating) of images morphed to the *same sex* (20%–100%) was significantly (*Z* > 2.3, *p* < .05, corrected) associated with activation in the precuneus and bilateral middle frontal gyri only in females. There were no significant associations for “not me” ratings in males. Participants' ratings of less self‐similarity (greater “not me” rating) when viewing opposite‐sex other bodies were significantly (*Z* > 2.3, *p* < .05, corrected) associated with activation in the bilateral TPJ and precuneus in both men and women. Men in addition showed significantly (*Z* > 2.3, *p* < .05, corrected) associated greater activations in the vmPFC and bilateral anterior temporal gyri. When males and females were directly compared regarding associations to greater “not me” ratings, males had significantly (*Z* > 2.3, *p* < .05, corrected) stronger associations in the bilateral amygdalae, precuneus, and posterior cingulate (Table 3).

**Figure 5:**
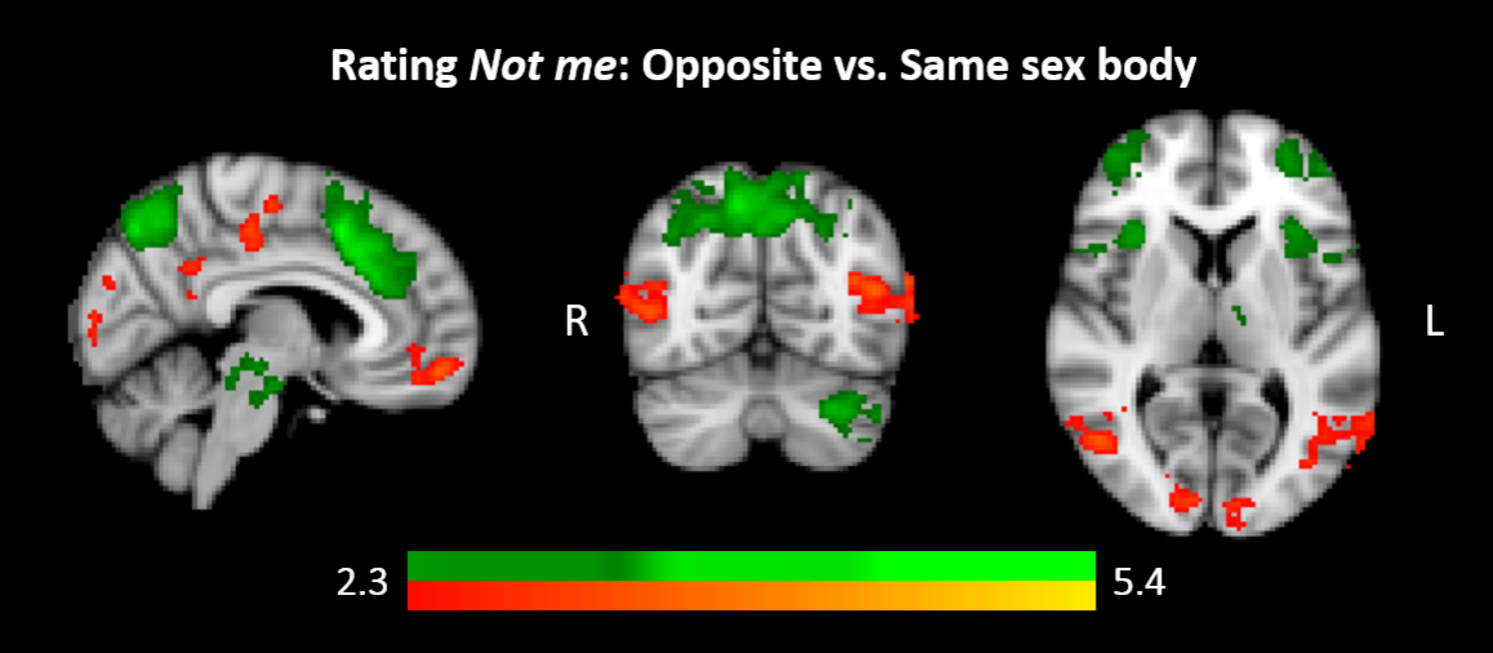
Across male and female participants, parametrically‐modeled “not me” ratings when viewing images of same sex and opposite sex other bodies of different morph degrees; red color = activation for the contrast “opposite sex – same sex bodies rated as ‘not me’”; green color = activation for the contrast “same sex – opposite sex bodies rated as ‘not me’”; MNI coordinates of the slices shown: *x* = 6, *y* = −66, *z* = 6; R = right, L = left; color bars indicate *z* value of the presented contrast

As noted above, brain regions that were associated with participants' ratings of self‐similarity (whether greater “me” or “not me” rating) differed when participants were viewing either the opposite or same‐sex—suggesting that the activation could be perceptually driven. To further investigate this possibility, we directly contrasted viewing of opposite versus same sex images in a combined group of males and females when parameterized to greater “not me” rating. Here, greater *“not me”* ratings when viewing bodies of the *same sex* versus the opposite sex were significantly (*Z* > 2.3, *p* < .05, corrected) associated with activation in the bilateral insula, bilateral vlPFC, right dlPFC, anterior cingulate cortices, left thalamus, and left cerebellum. By contrast, greater *“not me”* ratings when viewing bodies of the *opposite sex* versus the same sex were significantly (*Z* > 2.3, *p* < .05, corrected) associated with activation in the bilateral lateral occipital cortex (EBA), precuneus/posterior cingulate cortex, vmPFC, and left‐FBA, providing further evidence that the pattern of activation could be perceptually driven (Table 4 and Figure 5).

**Table 4:** Brain activation for parametrically modeled greater “not me” rating while viewing images morphed to the same versus opposite sex

Contrast	*Z*‐cluster threshold	Region	Side	*x*	*y*	*z*	*Z* max	Cluster size^a^
Same‐sex > opposite‐sex	2.3	Paracingulate gyrus	R	6	14	46	5.45	8,623
		Middle frontal gyrus	R	44	34	18	5.25	
		Insular cortex and frontal orbital cortex	R	36	24	−2	5.11	
		Precuneus cortex	R	10	−62	50	4.94	4,239
		Supramarginal gyrus, posterior division	R	46	−36	42	4.7	
		Insular cortex	L	−30	24	0	4.93	1810
		Middle frontal gyrus	L	−50	22	28	4.08	
		Inferior frontal gyrus	L	−38	18	20	3.82	
		Frontal operculum cortex	L	−40	16	2	3.69	
		Brainstem	R	4	−14	−18	3.49	831
		Thalamus	L	−6	−10	−4	3.33	
		Frontal pole	L	−46	44	−12	3.5	591
		Cerebellum	L	−30	−68	−34	3.82	543
Opposite‐sex > same‐sex	2.3	Lateral occipital cortex	L	−18	−86	26	4.16	2,479
		Angular gyrus	L	−44	−58	14	3.75	
		Cuneal cortex	L	−12	−86	32	3.75	
		Posterior cingulate	L	−10	−42	36	3.72	1,651
		Postcentral gyrus	R	42	−26	62	3.44	
		Frontal medial cortex		0	52	−14	3.85	814
		Frontal pole	L	−2	62	−8	3.51	
		Paracingulate gyrus	R	2	46	−2	3.39	
		Lateral occipital cortex, superior division	R	24	−84	32	4.38	787
		Occipital pole	R	16	−92	16	3.58	
		Lateral occipital cortex, inferior division and middle temporal gyrus, temporooccipital part	R	62	−60	12	4	746
		Lateral occipital cortex, superior division and angular gyrus	R	50	−60	16	3.83	
		Supramarginal gyrus	L	−66	−46	32	3.85	532
		Parietal operculum cortex	L	−58	−38	24	3.3	
		Temporal (occipital) fusiform cortex, posterior division	L	−28	−42	−16	3.88	499
		Lingual gyrus	L	−26	−52	−8	3.71	
		Parahippocampal gyrus, posterior division	L	−24	−36	−18	3.21	

a Cluster size is number of voxels with voxel size of 3 × 3 × 3.5 mm; R = right; L = left.

## 4 DISCUSSION

The current study investigated whether cerebral processing of the perception of one's own body and of other bodies in the context of self differs between men and women. Perception of own, unmorphed bodies showed no sex differences, and involved activation of a set of brain regions previously described to be associated with perceptual recognition of self as well as during perceptual decisions about object identity (Ploran et al., 2007). This included body perception regions (EBA, FBA), and areas involved in self‐referential processing, such as the medial PFC, anterior insula, and thalamus (Amodio & Frith, 2006; D'Argembeau, 2013; D'Argembeau et al., 2007; Denny et al., 2012; Mitchell et al., 2006; Murray et al., 2012; van der Meer et al., 2010). Furthermore, activation of bilateral caudate nuclei was observed, congruent with previous reports about its involvement in processing of body and limb posture (Villablanca, 2010). Finally, there was deactivation of the precuneus, right temporal pole, and both TPJ‐regions known to be involved in self‐other distinction, mentalizing, and perspective taking (Eddy, 2016; Payne & Tsakiris, 2017; van der Cruijsen et al., 2017). We also found activation in the cerebellum during own body perception, which is in line with a study describing its inclusion in a neuronal network underlying illusory own‐body perceptions (Schutter, Kammers, Enter, & Van Honk, 2006).

In sum, own body perception in the context of self involves cerebral processes related to one's own body schema, identification of self, as well as the specific distinction and comparison of self from and with others. Importantly, these processes do not seem to differ between men and women.

Interestingly, and to the best of our knowledge not described earlier, during perception of *other bodies of the same* sex (contrasted to the *scrambled image*), men and women engaged very similar brain areas as when viewing their own body, including the EBA, FBA, bilateral caudate, thalamus, bilateral anterior cingulate cortices, bilateral anterior insula, ventrolateral PFC, and dorsal mPFC. This was true also for the deactivation pattern (TPJ, temporal pole), with the only exception that it was more right‐lateralized in women than in men (Eddy, 2016).

One possible explanation for this similarity is that self‐referential information may be experienced and generalized to others who look similar to us (Platek, Krill, & Kemp, 2008; Tsakiris, 2017). It was also suggested that coactivation of the reward system and the dorsal anterior cingulate cortices during evaluation of self compared to others might contribute to the integration of social comparisons into evaluation of self (Lindner et al., 2015). Interestingly, an fMRI study (Lübke et al., 2014) that used body *odors* rather than visual body stimuli found very similar brain regions involved during perception of others' (males and females) body odors—the fusiform cortex, the anterior and posterior cingulate cortices, and the anterior insular cortex.

As opposed to the “own, unmorphed body” condition, viewing another body of the same sex revealed a sex difference, with men having a more pronounced activation than women in the left lateral occipital cortex, which could be an indication of heightened attention towards same‐sex others. There was also a sex difference in the precuneus cortex, due to greater *de*activation of this region in female participants, implying that women might have less of self and same sex other differentiation compared to men (see further discussion).

Notably, these sex differences in *same‐sex other* body perception became even more apparent when *contrasted to the own body* (0% morphed, rather than scrambled image). Whereas in female participants there was no significant difference in brain activation during own body and same‐sex other body perception, (there was a stronger *de*activation of the EBA), in male participants there was an increased activation of the FBA, left precentral gyrus and left ventrolateral PFC–when viewing another same sex body compared to the own body. A recent study showed that these latter brain areas were involved in decoding familiarity (of faces, bodies, and gait) (Hahn & O'Toole, 2017). It may thus be possible that men show increased engagement, together with higher attentional load, in cognitive decision processes on differentiating between self and same‐sex others. This potentially could be evoking intrasexual competition (Buunk & Massar, 2012) and/or could help to discern what is related and similar as opposed to different from self. Moreover, the sex differences in neural activations during perception of bodies similar to one's own may indicate that women more easily adopt other female bodies as “self” than men. This is also supported by the observed cerebellar activations during own and same‐sex other perception specifically in females, which have been reported to be involved in illusory own body perception (Schutter et al., 2006). Thus, our findings may be interpreted as that women may more easily be able to put themselves in other females' shoes, which require Theory of Mind (ToM), the ability to explain and predict other people's mental states, and cognitive empathy. Indeed, several previous studies have suggested sex differences in mind reading abilities as well as empathy (Adenzato et al., 2017; Frank, Baron‐Cohen, & Ganzel, 2015; Krach et al., 2009; Schulte‐Rüther, Markowitsch, Shah, Fink, & Piefke, 2008; Singer & Lamm, 2009).

A third major observation in this study related to perception of bodies of the opposite sex. When compared to the *scrambled image*, both men and women activated general as well as body perception‐specific attention circuits (EBA, FBA, right‐dorsolateral PFC). However, and notably, sex differences were most pronounced when contrasting viewing bodies of an opposite sex other to the *own body* (unmorphed image). The two groups differed distinctly in that men activated, whereas women *de*activated the precuneus and right TPJ. In addition, during viewing an opposite sex other body and when rating an image of their body that appeared female (morphed to the opposite sex) as “not me,” men showed activation in the visual cortex, caudate nucleus, precuneus, and bilateral amygdala, regions reported to be involved in other rather than self‐orientation (Bischoff et al., 2012; Eddy, 2016), sexual arousal (Ponseti et al., 2006), and emotional salience (Gerber et al., 2008; Phan et al., 2003).

Together, these data hint that the other body in relation to self might have a greater salience in men (van Hooff, Crawford, & van Vugt, 2011), whereas for women images of the own body are more salient. The observed sex differences may have implications when trying to understand conditions involving own body perceptions such as anorexia nervosa, gender dysphoria, or autism spectrum disorders, which all show a sex skewed prevalence. Females previously were found to be more sensitive to information about their own body than males (Mitchison et al., 2017; Powell & Hendricks, 1999), and therefore perhaps have a less distinct or a more vulnerable own body schema, rendering them more prone to internalized distorted perceptions of their own bodies. Females may also easier adopt other females' bodies as “self” and, conversely, do not accept the image of one's body as “self.” Worth mentioning is that all the participants were heterosexual, thus the discussion only pertains to heterosexual cis‐gender persons.

In addition to investigating whether men and women engage different cerebral networks during perception of own and other bodies in the context of self, we also approached this at a different level: when distinguishing self from others, does the brain show differences depending on whether it is viewing the same or the opposite sex? To investigate this, we directly contrasted rating “not me” of same sex versus rating “not me” of opposite sex bodies. Here, greater *“not me”* ratings when viewing *same sex* bodies compared with opposite sex bodies was significantly associated with activation in regions involved in (illusory) own body perception and comparative processes (Kedia, Mussweiler, & Linden, 2014). Yet, the same *“not me”* ratings but when viewing *opposite sex* bodies compared with same sex others did engage (body) perceptual and evaluative regions (Kedia et al., 2014). This suggests that the activations were dominated by perceptual—the type of visual body stimuli—rather than cognitive processes, since the latter was same in both cases: rating “not me.”

Interestingly, and in support of this notion, a neuroimaging study using body odor stimuli from either the sisters or same‐sex best friends of a group of 12 women, showed that, independently of conscious recognition, olfactory‐based kin recognition activated self‐referential brain regions when smelling body odors of their sisters as compared to their female friends (Lundström, Boyle, Zatorre, & Jones‐Gotman, 2009). In that study, kin recognition, via the mechanism of so‐called “automatic self‐referent phenotype matching” (Mateo & Johnston, 2000), recruited self‐referential networks without any cognitive or conscious identification process involved. Together with the current study, these observations suggest that sensory body perception (visual or olfactory) seems to overrule cognitive perception (i.e., labeling a given body as “me” or “not me”), which was previously shown for other stimuli of high social and ecological importance, such as body odors, emotional faces, and infant crying and laughing sounds (Lundström, Boyle, Zatorre, & Jones‐Gotman, 2008; Morris, Öhman, & Dolan, 1998; Seifritz et al., 2003). Whether this overruling of sensory over cognitive perception also applies to other stimuli remains to be further investigated.

Our findings should be viewed in light of its limitations. First, we did not assess participants' impression of the body stimuli afterward outside the scanner in terms of how attractive the opposite‐sex, or same‐sex body stimuli were perceived. It is possible that the male participants considered the opposite sex stimuli as more attractive than did the female participants, which might partially explain our findings of stronger attention and reward‐related brain activation in men for this condition. In addition, this information would have helped to establish more direct links between the activation patterns and cognitive/evaluative processes other than the subjective degree that the body was similar to theirs, about which we could only make post hoc inferences. We also did not obtain any ratings from independent raters of how similar the morph‐to stimuli bodies were to the participants' bodies and did not measure participants' body weight or body mass index. It may have theoretically been possible, by chance, that the female morph‐to bodies used were better comparable, in terms of, for example, height, weight, shape, or muscularity, to those of the female participants than how the male morph‐to bodies compared to the male participants' bodies. This might have affected the sex differences we observed in the same‐sex other versus own body condition. However, this is mitigated partially by the fact that based on the investigators' subjective impression, none of our participants had extremely different body composition than the morph‐to stimuli bodies; for example, none appeared obese or extremely underweight. Finally, though only (self‐reported) healthy participants were included, we did not perform a structured assessment of any prior or current eating disorder (or other psychiatric disorders). Therefore, we cannot rule out that there may have been (if the participants were unaware or did not report accurately) any disturbances in body image or possible concerns about the own body, that might have resulted in own‐body stimuli being much more emotionally salient and that might have been more common in one of the groups.

In conclusion, we provide first evidence that the neural representation of own body does not differ appreciably between the sexes. In contrast, perception of other bodies, in particular of the opposite sex, could be a particularly salient social signal to men, whereas for women the own body likely has higher relevance.

## CONFLICT OF INTERESTS

The authors have no conflict of interest.

## Supporting information

Figure S1

Supinfo. Supplementary Material.
