# Supplementary figures and images for: Sex differences in own and other body perception
Source: Hum Brain Mapp. 2018 Nov 15;40(2):474–88. doi: 10.1002/hbm.24388 (PMC6587810; doi:10.1002/hbm.24388)

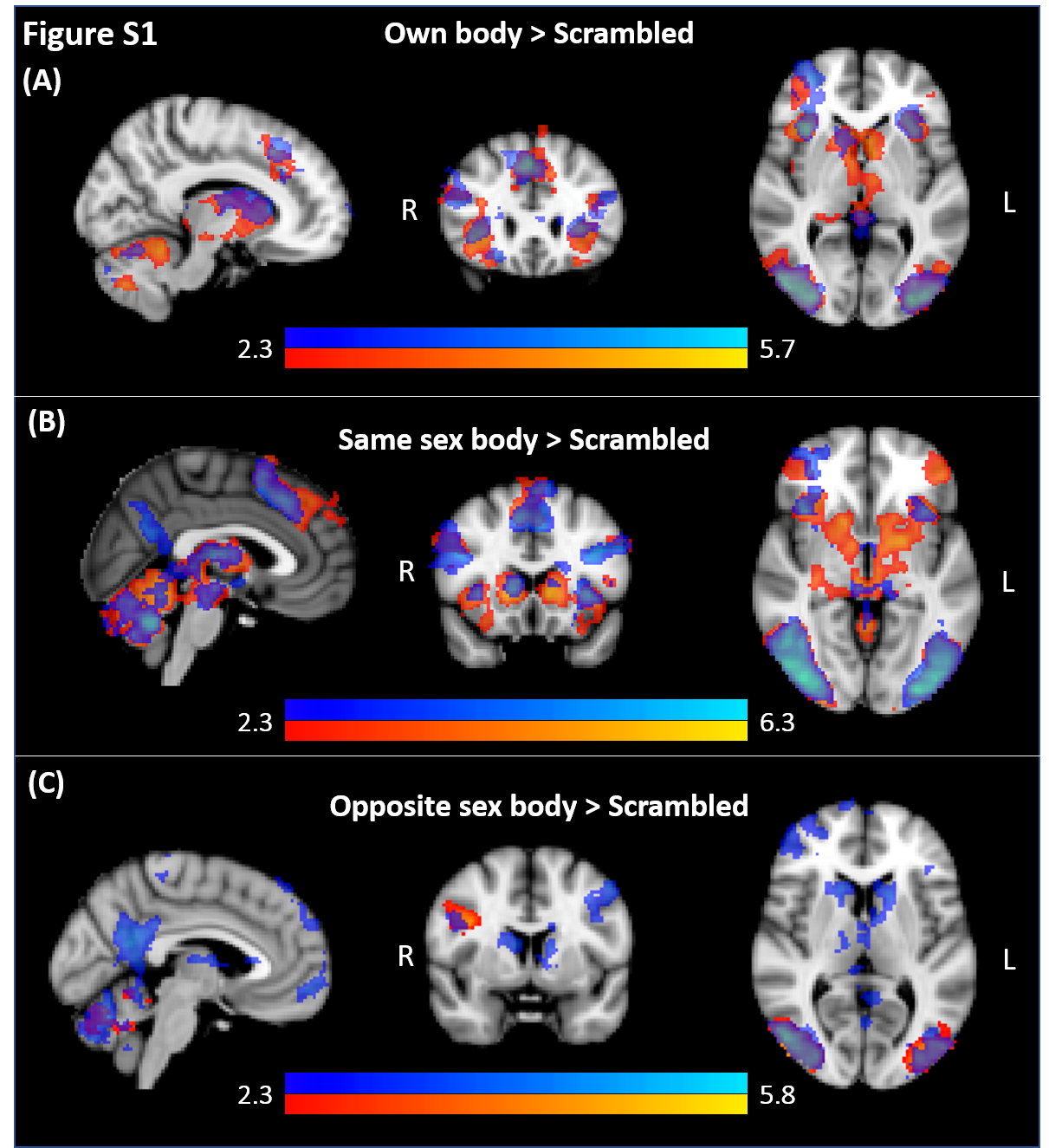

Supplement: Supplementary file 1 — Figure S1 [file HBM-40-474-s001.tif]
