# Supplementary material for: Sex differences in own and other body perception
Source: Hum Brain Mapp. 2018 Nov 15;40(2):474–88. doi: 10.1002/hbm.24388 (PMC6587810; doi:10.1002/hbm.24388)
Supplement: Supplementary file 2 — Supinfo. Supplementary Material. [file HBM-40-474-s002.docx]

**Supplementary Material**

**Results**

*Body localizer task*

The contrast *bodies – chairs* of the body localizer task resulted in significant (*Z* > 2.3, *p* < .05, corrected) bilateral activation in areas specialized for body perception, in both males and females. These areas included bilateral lateral occipital cortices, temporal occipital fusiform gyri, precuneus, left angular gyrus, bilateral precentral gyri, and the right amygdala in men. Both groups also showed deactivations in the fusiform cortex for this contrast (Table S1).

*Short 0.5s presentation time*

Results from trials of the long 2s duration are presented in the main text, the results from the short 0.5s image presentation trials can be found below.

Self-perception indices for the short condition trials were positive for both groups, indicating, as reported earlier (Feusner, Dervisic, et al., 2016), self-identification for images morphed to the same sex. Males’ and females’ ratings of self-perception indices did not differ significantly (Females: *M*=31.6, *SE*=4.8; Males: *M*=38.9, *SE*=4.7; *T*=1.1(28); *p*=.284).

*Own body perception*

In the short image presentation condition (0.5s), contrasting perception of one’s own body (0% morph) with the scrambled image baseline revealed significant (Z > 2.3, p < .05, corrected) activation in both groups in paracingulate cortex, right frontal pole, and left sensorimotor cortex. In addition, men showed activation in the right inferior frontal cortex and bilateral extrastriate body area. Both groups showed deactivation in the hippocampus, though this was right sided in males and bilateral in females. Females additionally showed greater deactivation in the posterior cingulate, precuneus, and bilateral TPJ (middle temporal gyri, angular gyri, and supramarginal gyri) (Table S2).

There were no significant differences in activation between men and women when contrasting perception of the own body (0% Morph) with the scrambled image baseline.

*Same sex other body - scrambled image*

In the short (0.5s) viewing condition, both groups showed significant activation (*Z* > 2.3, *p* < .05, corrected) in the bilateral inferior occipital cortices, paracingulate cortex, insula (left sided in females and right-sided in males), and the middle frontal gyrus (bilateral in females, right-sided in males). Females also showed bilateral caudate nucleus involvement. Both groups showed deactivation in the TPJ (bilateral in females, right-sided in males). When comparing males and females, females showed significantly greater activation (*Z* > 2.3, *p* < .05, corrected) in the bilateral superior frontal gyrus and frontal pole (Table S3).

*Same sex other body – Own body*

In contrast to the long (2s) viewing condition in which only males showed significant activation, only females showed significant activation in the short (0.5s) viewing condition. Females showed significant activation (*Z* > 2.3, *p* < .05, corrected) in the bilateral superior lateral occipital cortex, bilateral frontal pole, posterior cingulate, and precuneus (all of which were also significant when directly comparing females with males). In addition, females showed greater activation in the right amygdala (though this did not remain significant in the direct group contrast) (Table S4).

*Opposite sex other body - scrambled image*

In the short (0.5s) viewing condition, there were no significant group differences between males and females. Both groups had significant (*Z* > 2.3, *p* < .05, corrected) lateral occipital cortex activation (bilateral in males, right-sided in females). Females showed bilateral TPJ deactivation, and both groups showed deactivation in medial visual regions (Table S5).

*Opposite sex other body – Own body*

In the short (0.5s) viewing condition, females showed greater activation (*Z* > 2.3, *p* < .05, corrected) in the bilateral lateral occipital cortex, precuneus, posterior cingulate, and bilateral parahippocampal gyrus. Activation in males was limited to the frontal pole. Both groups showed deactivation in the paracingulate gyrus. When compared directly, females showed greater activation than males in the precuneus cortex (Table S6).

*Rating-dependent same sex body and opposite sex body perception*

Participants’ responses to the question “To what degree is this picture you?” when viewing *any* *morphed* image (images morphed from 20-100%, excluding the unmorphed image of self) were parametrically modeled on a scale from 1 to 4 (see description of *Body Perception Task* in the main text).

In the short viewing condition, when viewing images morphed to the *opposite sex* (20-100%), participants’ ratings of greater self-similarity (greater “me” rating) was significantly (*Z* > 2.3, *p* < 0.05, corrected) associated with activation in the right superior and middle frontal gyri, only in men (Table S8). Images morphed to the same sex did not reveal any significant activations associated with rating “me” or “not me” (Table S7). There were no significant group differences for any of the contrasts.

**Figure caption**

**Figure S1** Brain activation in men (blue-light blue colour) and women (red-yellow colour) when viewing (A) images of the own body, (B) a same sex other body, or (C) an opposite sex other body, contrasted to a scrambled control image, respectively; MNI coordinates of the slices shown: (A) x=12, y=26, z=2; (B) x=2, y=16, z=-4; (C) x=-4, y=4, z=6; R = right, L = left; colour bars indicate z-value of the presented contrast;

**Table S1** Sex differences in brain (de)activation during the Body Localizer Task, for the contrast Bodies > Chairs

| **Group contrast** | **Region** | **Side** | **x** | **y** | **z** | **Z-max** | **Size (vox)** |
| --- | --- | --- | --- | --- | --- | --- | --- |
| Males > Females | Postcentral Gyrus | R | 36 | -32 | 44 | 3.33 | 843 |
|  | Precentral Gyrus | R | 34 | -26 | 60 | 3 |  |
|  | Superior Frontal Gyrus | R | 22 | 2 | 66 | 2.99 |  |
|  | Precentral Gyrus, Superior Frontal Gyrus | L | -30 | -4 | 68 | 3.54 | 551 |
|  | Middle Frontal Gyrus | L | -28 | -2 | 58 | 3.23 |  |
| Females > Males | No Sig. |  |  |  |  |  |  |
| Females (Activation) | Lateral Occipital Cortex | R | 58 | -68 | 6 | 4.36 | 1902 |
|  | Middle Temporal Gyrus, temporooccipital part | R | 44 | -52 | 6 | 3.5 |  |
|  | Lateral Occipital Cortex | L | -54 | -70 | 14 | 4.47 | 1422 |
|  | Precuneus Cortex | L | -4 | -74 | 30 | 3.42 | 668 |
|  | Intracalcarine Cortex | R | 8 | -66 | 16 | 2.88 |  |
|  | Temporal (Occipital) Fusiform Cortex | R | 44 | -48 | -26 | 4.34 | 491 |
|  | Inferior Temporal Gyrus, temporooccipital part | R | 46 | -40 | -20 | 3.45 |  |
|  | Inferior Frontal Gyrus | L | -40 | 10 | 26 | 3.82 | 487 |
|  | Middle Frontal Gyrus | L | -52 | 16 | 40 | 3.13 |  |
| Males (Activation) | Precentral Gyrus, Superior Frontal Gyrus | L | -30 | -4 | 66 | 4.25 | 8515 |
|  | Middle Frontal Gyrus | L | -36 | -2 | 62 | 3.93 |  |
|  | Parietal Operculum Cortex | R | 58 | -30 | 24 | 3.9 |  |
|  | Inferior Frontal Gyrus, pars opercularis | L | -54 | 14 | 28 | 3.86 |  |
|  | Paracingulate Gyrus | L | -2 | 30 | 40 | 3.8 |  |
|  | Amygdala | R | 18 | -4 | -18 | 4.24 | 5854 |
|  | Thalamus | L | -4 | -24 | 10 | 3.87 |  |
|  | Accumbens | L | -10 | 4 | -6 | 3.75 |  |
|  | Putamen | L | -18 | 8 | 0 | 3.69 |  |
|  | Lateral Occipital Cortex | R | 50 | -66 | -2 | 4.88 | 4357 |
|  | Temporal Occipital Fusiform Cortex | R | 44 | -46 | -24 | 4.53 |  |
|  | Lateral Occipital Cortex | L | -54 | -70 | 4 | 5.19 | 3521 |
|  | Precuneus Cortex | R | 2 | -62 | 24 | 3.98 | 2166 |
|  | Cingulate Gyrus, posterior division | L | -4 | -38 | 26 | 3.32 |  |
|  | Frontal Pole | L | -28 | 62 | 4 | 3.79 | 1232 |
|  | Cerebellum | L | -14 | -74 | -30 | 3.69 | 1017 |
|  | Cerebellum | R | 6 | -84 | -28 | 3.38 | 501 |
|  | Frontal Medial Cortex | L | -6 | 48 | -18 | 3.36 | 451 |
|  | Frontal Medial Cortex | R | 6 | 48 | -18 | 3.17 |  |
| Females (Deactivation) | Temporal Occipital Fusiform Cortex | R | 30 | -42 | -16 | 4.81 | 8759 |
|  | Occipital Pole, Temporal Fusiform Cortex | L | -12 | -96 | -4 | 4.71 |  |
| Males (Deactivation) | Temporal Occipital Fusiform Cortex | L | -26 | -54 | -14 | 5.42 | 2749 |
|  | Temporal Occipital Fusiform Cortex | R | 28 | -56 | -12 | 5.16 | 2516 |

**Table S2** Brain (de)activation in men and women for the contrast Own body (0% Morph) > Scrambled image (control condition)

| Presentation  Time | Group contrast | Region | Side | x | y | z | Z-max | Size (vox) |
| --- | --- | --- | --- | --- | --- | --- | --- | --- |
| 2s (Long) | Females > Males | No sig. |  |  |  |  |  |  |
|  | Males > Females | No sig. |  |  |  |  |  |  |
|  | Females (activation) | Lateral Occipital Cortex, inferior division | R | 38 | -80 | -2 | 5.73 | 19905 |
|  |  | Postcentral Gyrus | L | -42 | -26 | 64 | 4.69 | 3881 |
|  |  | Supramarginal Gyrus, anterior division | L | -46 | -32 | 40 | 4.21 |  |
|  |  | Superior Parietal Lobule | L | -28 | -54 | 46 | 4.13 |  |
|  |  | Lateral Occipital Cortex, superior division | R | 26 | -60 | 40 | 4.94 | 2403 |
|  |  | Supramarginal Gyrus, posterior division | R | 44 | -36 | 46 | 4.79 |  |
|  |  | Superior Parietal Lobule | R | 30 | -52 | 40 | 4.43 |  |
|  |  | Paracingulate Gyrus | R | 6 | 26 | 42 | 4.46 | 1869 |
|  |  | Superior Frontal Gyrus | R | 2 | 42 | 38 | 3.52 |  |
|  |  | Precentral Gyrus | R | 46 | 8 | 30 | 5.54 | 975 |
|  |  | Insular Cortex | R | 42 | 0 | 6 | 3.48 |  |
|  |  | Central Opercular Cortex | R | 40 | -4 | 14 | 3.46 |  |
|  | Males (activation) | Occipital Pole | L | -32 | -92 | -4 | 5.21 | 14349 |
|  |  | Lateral Occipital Cortex, inferior division | L | -34 | -88 | 4 | 4.9 |  |
|  |  | Middle Frontal Gyrus | R | 48 | 32 | 22 | 4.87 |  |
|  |  | Lateral Occipital Cortex, inferior division | R | 48 | -76 | -8 | 5.71 | 5864 |
|  |  | Inferior Temporal Gyrus, temporooccipital part | R | 50 | -56 | -6 | 5.1 |  |
|  |  | Paracingulate Gyrus | R | 4 | 26 | 46 | 4.56 | 1030 |
|  |  | Superior Frontal Gyrus | L | -2 | 12 | 58 | 3.63 |  |
|  |  | Juxtapositional Lobule Cortex (formerly Supplementary Motor Cortex) | L | -2 | 8 | 62 | 3.45 |  |
|  | Females (deactivation) | Occipital Pole | L | -14 | -92 | 14 | 5.19 | 5009 |
|  |  | Lingual Gyrus, Temporal Occipital Fusiform Cortex | L | -26 | -56 | -6 | 4.83 |  |
|  |  | Lateral Occipital Cortex, superior division | L | -18 | -88 | 26 | 4.35 |  |
|  |  | Angular Gyrus | R | 56 | -50 | 24 | 5.33 | 4444 |
|  |  | Middle Temporal Gyrus, temporooccipital part | R | 64 | -44 | 10 | 4.92 |  |
|  |  | Supramarginal Gyrus, posterior division | R | 64 | -40 | 30 | 4.79 |  |
|  |  | Middle Temporal Gyrus, temporooccipital part | L | -64 | -48 | 2 | 4.68 | 2849 |
|  |  | Supramarginal Gyrus | L | -60 | -38 | 52 | 4.45 |  |
|  |  | Angular Gyrus | L | -48 | -50 | 24 | 4.42 |  |
|  |  | Cingulate Gyrus, posterior division | R | 10 | -32 | 46 | 4.79 | 2134 |
|  |  | Precuneous Cortex | R | 10 | -36 | 46 | 4.68 |  |
|  | Males (deactivation) | Occipital Pole | R | 12 | -90 | 16 | 4.66 | 3876 |
|  |  | Occipital Fusiform Gyrus | R | 26 | -66 | -8 | 4.5 |  |
|  |  | Lingual Gyrus, Temporal Occipital Fusiform Cortex | R | 26 | -56 | -8 | 4.39 |  |
|  |  | Angular Gyrus, Middle Temporal Gyrus, temporooccipital part | R | 52 | -48 | 14 | 4.61 | 3690 |
|  |  | Supramarginal Gyrus, posterior division | R | 58 | -46 | 34 | 4.51 |  |
|  |  | Angular Gyrus | L | -60 | -54 | 14 | 4.73 | 1103 |
|  |  | Supramarginal Gyrus, posterior division | L | -58 | -44 | 18 | 3.59 |  |
| 0.5s (Short) | Females > Males | No sig. |  |  |  |  |  |  |
|  | Males > Females | No sig. |  |  |  |  |  |  |
|  | Females (activation) | Postcentral Gyrus, Precentral Gyrus | L | -40 | -28 | 56 | 5.16 | 1947 |
|  |  | Paracingulate Gyrus | L | -2 | 22 | 46 | 4.36 | 1645 |
|  |  | Cerebellum | R | 20 | -48 | -24 | 4.23 | 1485 |
|  |  | Frontal Pole | R | 46 | 36 | 20 | 3.72 | 593 |
|  | Males (activation) | Frontal Pole | R | 32 | 56 | 14 | 4.23 | 1529 |
|  |  | Paracingulate Gyrus | R | 6 | 18 | 44 | 4.25 | 1393 |
|  |  | Superior Frontal Gyrus | R | 8 | 10 | 56 | 3.36 |  |
|  |  | Frontal Orbital Cortex and Operculum | R | 32 | 28 | 4 | 4.64 | 777 |
|  |  | Inferior Frontal Gyrus, pars opercularis | R | 50 | 16 | 8 | 2.72 |  |
|  |  | Postcentral Gyrus | L | -46 | -32 | 50 | 3.44 | 694 |
|  |  | Lateral Occipital Cortex, superior division | L | -22 | -64 | 60 | 3.22 |  |
|  |  | Superior Parietal Lobule | L | -28 | -58 | 56 | 3.14 |  |
|  |  | Lateral Occipital Cortex, inferior division | L | -46 | -78 | 2 | 3.62 | 627 |
|  |  | Inferior Temporal Gyrus, temporooccipital part | R | 52 | -56 | -8 | 3.68 | 603 |
|  |  | Lateral Occipital Cortex, inferior division | R | 48 | -76 | -4 | 3.66 |  |
|  | Females (deactivation) | Angular Gyrus | R | 52 | -48 | 26 | 4.78 | 7242 |
|  |  | Middle Temporal Gyrus | R | 58 | -6 | -16 | 4.27 |  |
|  |  | Lateral Occipital Cortex, superior division | R | 44 | -62 | 24 | 4.2 |  |
|  |  | Superior Temporal Gyrus, anterior division | R | 58 | 0 | -12 | 4.12 |  |
|  |  | Cingulate Gyrus, posterior division | R | 8 | -46 | 36 | 4.37 | 4907 |
|  |  | Precuneous Cortex and Cingulate Gyrus, posterior division | R | 8 | -38 | 46 | 4.17 |  |
|  |  | Superior Temporal Gyrus | L | -66 | -32 | 0 | 4.22 | 2271 |
|  |  | Lingual Gyrus and Temporal/Occipital Fusiform Cortex | L | -26 | -54 | -8 | 4.37 | 1734 |
|  |  | Left Hippocampus | L | -28 | -26 | -14 | 3.97 |  |
|  |  | Parahippocampal Gyrus, posterior and Temporal/Occipital Fusiform Cortex | R | 26 | -36 | -16 | 4.68 | 1600 |
|  |  | Right Hippocampus | R | 26 | -20 | -14 | 4.01 |  |
|  |  | Angular Gyrus | L | -56 | -58 | 26 | 4.36 | 1474 |
|  |  | Supramarginal Gyrus, posterior division | L | -54 | -50 | 34 | 4.29 |  |
|  |  | Lateral Occipital Cortex, superior division | L | -44 | -70 | 34 | 3.52 |  |
|  |  | Frontal Pole and Frontal Medial Cortex |  | 0 | 54 | -6 | 3.43 | 725 |
|  |  | Frontal Medial Cortex | R | 6 | 54 | -10 | 3.41 |  |
|  |  | Paracingulate Gyrus | L | -12 | 48 | -4 | 3.16 |  |
|  |  | Frontal Pole |  | 0 | 62 | -2 | 2.97 |  |
|  | Males (deactivation) | Superior Temporal Gyrus, anterior division | R | 58 | 0 | -10 | 3.7 | 567 |
|  |  | Middle Temporal Gyrus | R | 56 | -2 | -28 | 3.38 |  |
|  |  | Lingual Gyrus and Temporal Occipital Fusiform Cortex | R | 32 | -50 | -4 | 3.55 | 504 |
|  |  | Right Amygdala and Right Hippocampus | R | 22 | -8 | -16 | 2.98 |  |

**Table S3** Sex differences in brain (de)activation for the contrast Same Sex other body (80-100% morphed) > Scrambled image (control condition)

| Presentation  Time | Group contrast | Region | Side | x | y | z | Z-max | Size (vox) |
| --- | --- | --- | --- | --- | --- | --- | --- | --- |
| 2s (Long) | Males > Females | Lateral Occipital Cortex, superior division | L | -28 | -78 | 22 | 3.54 | 501 |
|  |  | Cuneal Cortex, Precuneous Cortex | L | -20 | -70 | 28 | 3.14 |  |
|  |  | Occipital Pole | L | -24 | -90 | 26 | 3.1 |  |
|  | Females > Males | No Sig. |  |  |  |  |  |  |
|  | Females (activation) | Lateral Occipital Cortex, inferior division | R | 40 | -82 | -6 | 6.38 | 32547 |
|  |  | Precentral Gyrus, Inferior Frontal Gyrus, pars opercularis | R | 40 | 10 | 28 | 5.76 |  |
|  |  | Supramarginal Gyrus | R | 44 | -36 | 46 | 5.42 | 3575 |
|  |  | Lateral Occipital Cortex, superior division | R | 26 | -60 | 42 | 5.1 |  |
|  |  | Paracingulate Gyrus | L | -6 | 28 | 40 | 4.58 | 2547 |
|  |  | Frontal Pole | L | -8 | 56 | 42 | 3.87 |  |
|  |  | Middle Frontal Gyrus | R | 28 | 4 | 50 | 3.68 | 461 |
|  |  | Precentral Gyrus | R | 26 | -6 | 50 | 3.63 |  |
|  |  | Superior Frontal Gyrus | R | 28 | 0 | 62 | 2.98 |  |
|  | Males (activation) | Lateral Occipital Cortex, inferior division | L | -44 | -78 | -12 | 6.04 | 11480 |
|  |  | Occipital Pole | L | -30 | -92 | -6 | 5.19 |  |
|  |  | Temporal Occipital Fusiform Cortex | L | -40 | -48 | -16 | 5.14 |  |
|  |  | Occipital Fusiform Gyrus | L | -40 | -70 | -12 | 5.04 |  |
|  |  | Lateral Occipital Cortex, inferior division | R | 44 | -62 | -12 | 5.7 | 8649 |
|  |  | Superior Parietal Lobule | R | 34 | -46 | 52 | 5.47 |  |
|  |  | Supramarginal Gyrus, anterior division | R | 62 | -20 | 40 | 5.19 |  |
|  |  | Frontal Pole | R | 42 | 38 | 8 | 4.85 | 4106 |
|  |  | Middle Frontal Gyrus | R | 44 | 34 | 22 | 4.75 |  |
|  |  | Insular Cortex | R | 38 | 20 | 0 | 4.59 |  |
|  |  | Precentral Gyrus | R | 50 | 10 | 24 | 4.32 |  |
|  |  | Inferior Frontal Gyrus, pars opercularis | R | 46 | 16 | 24 | 4.17 |  |
|  |  | Inferior Frontal Gyrus, pars opercularis | L | -36 | 18 | 24 | 4.5 | 2663 |
|  |  | Middle Frontal Gyrus | L | -42 | 18 | 28 | 4.46 |  |
|  |  | Insular Cortex | L | -32 | 16 | 2 | 4.31 |  |
|  |  | Paracingulate Gyrus | L | -4 | 22 | 42 | 4.56 | 2009 |
|  |  | Superior Frontal Gyrus |  | 0 | 12 | 58 | 4.25 |  |
|  |  | Postcentral Gyrus, Supramarginal Gyrus, anterior division | L | -48 | -34 | 50 | 4.1 | 1696 |
|  |  | Superior Parietal Lobule | L | -34 | -50 | 56 | 3.89 |  |
|  |  | Lateral Occipital Cortex | L | -24 | -66 | 60 | 3.6 |  |
|  | Females (deactivation) | Lateral Occipital Cortex, superior division | L | -16 | -88 | 18 | 5.34 | 6445 |
|  |  | Occipital Pole | L | -12 | -92 | 12 | 5.33 |  |
|  |  | Lateral Occipital Cortex, superior division | R | 16 | -86 | 26 | 5.22 |  |
|  |  | Angular Gyrus | R | 60 | -48 | 22 | 5.61 | 5742 |
|  |  | Supramarginal Gyrus, posterior division | R | 66 | -40 | 30 | 4.8 |  |
|  |  | Middle Temporal Gyrus, temporooccipital part | R | 50 | -38 | 4 | 4.53 |  |
|  |  | Angular Gyrus | L | -62 | -56 | 12 | 5.31 | 4602 |
|  |  | Supramarginal Gyrus, posterior division | L | -50 | -48 | 24 | 4.61 |  |
|  |  | Middle Temporal Gyrus, temporooccipital part | L | -68 | -52 | 2 | 4.59 |  |
|  |  | Precuneous Cortex and Cingulate Gyrus, posterior division | R | 6 | -36 | 48 | 5.54 | 3031 |
|  |  | Paracingulate Gyrus | R | 14 | 48 | 2 | 3.64 | 498 |
|  |  | Cingulate Gyrus, anterior division | L | -2 | 40 | 2 | 3 |  |
|  |  | Frontal Pole | L | -8 | 56 | -4 | 2.91 |  |
|  | Males (deactivation) | Angular Gyrus | R | 54 | -46 | 24 | 4.72 | 3621 |
|  |  | Supramarginal Gyrus, posterior division | R | 56 | -40 | 20 | 4.35 |  |
|  |  | Middle Temporal Gyrus, temporooccipital part | R | 64 | -54 | 8 | 4.24 |  |
|  |  | Superior Temporal Gyrus, posterior division | R | 68 | -34 | 12 | 4.23 |  |
|  |  | Occipital Pole | R | 10 | -90 | 16 | 4.75 | 3168 |
|  |  | Lingual Gyrus | L | -12 | -78 | -8 | 4.59 |  |
|  |  | Angular Gyrus | L | -62 | -52 | 14 | 4.43 | 1095 |
|  |  | Supramarginal Gyrus, posterior division | L | -54 | -50 | 12 | 4.12 |  |
|  |  | Middle Temporal Gyrus, temporooccipital part | L | -56 | -50 | 4 | 3.87 |  |
| 0.5s (Short) | Females > Males | Frontal Pole | L | -14 | 46 | 44 | 3.38 | 750 |
|  |  | Superior Frontal Gyrus | R | 4 | 34 | 46 | 3.35 |  |
|  |  | Superior Frontal Gyrus | L | -6 | 28 | 46 | 3.15 |  |
|  | Males > Females | No Sig. |  |  |  |  |  |  |
|  | Females (activation) | Right Caudate | R | 14 | 18 | 4 | 4.47 | 8431 |
|  |  | Insular Cortex | L | -34 | 18 | 0 | 4 |  |
|  |  | Left Caudate | L | -10 | 12 | 8 | 3.95 |  |
|  |  | Paracingulate Gyrus | L | -4 | 34 | 32 | 4.4 | 4334 |
|  |  | Frontal Pole | R | 44 | 38 | 16 | 5.04 | 2467 |
|  |  | Middle Frontal Gyrus | R | 54 | 34 | 28 | 4.32 |  |
|  |  | Middle Frontal Gyrus | L | -48 | 8 | 40 | 3.72 | 1798 |
|  |  | Frontal Pole | L | -42 | 46 | 4 | 3.68 |  |
|  |  | Cerebellum | L | -36 | -72 | -40 | 4.74 | 1463 |
|  |  | Lateral Occipital Cortex, superior division | R | 34 | -62 | 52 | 4.42 | 1454 |
|  |  | Supramarginal Gyrus | R | 48 | -36 | 50 | 3.98 |  |
|  |  | Lateral Occipital Cortex, superior division | R | 30 | -68 | 54 | 3.69 |  |
|  |  | Precentral Gyrus | L | -38 | -20 | 56 | 3.61 | 988 |
|  |  | Postcentral Gyrus | L | -42 | -22 | 64 | 3.54 |  |
|  |  | Superior Parietal Lobule | L | -26 | -56 | 46 | 3.3 |  |
|  |  | Lateral Occipital Cortex | L | -30 | -60 | 48 | 3.17 |  |
|  | Males (activation) | Lateral Occipital Cortex, inferior division | L | -46 | -74 | -8 | 3.91 | 1850 |
|  |  | Superior Parietal Lobule | R | 34 | -50 | 50 | 4.37 | 1577 |
|  |  | Lateral Occipital Cortex, superior division | R | 32 | -64 | 46 | 3.78 |  |
|  |  | Frontal Operculum Cortex | R | 34 | 26 | 4 | 3.94 | 1571 |
|  |  | Middle Frontal Gyrus | R | 52 | 32 | 28 | 3.91 |  |
|  |  | Frontal Pole | R | 44 | 36 | 10 | 3.49 |  |
|  |  | Insular Cortex | R | 32 | 24 | -2 | 3.41 |  |
|  |  | Paracingulate Gyrus | L | -4 | 16 | 50 | 4.62 | 1024 |
|  |  | Inferior Temporal Gyrus, temporooccipital part | R | 52 | -56 | -6 | 4.4 | 999 |
|  |  | Lateral Occipital Cortex, inferior division | R | 50 | -76 | 0 | 4.21 |  |
|  |  | Temporal Occipital Fusiform Cortex | R | 38 | -40 | -20 | 3.3 |  |
|  |  | Superior Parietal Lobule | L | -34 | -52 | 54 | 3.65 | 912 |
|  |  | Lateral Occipital Cortex, superior division | L | -22 | -66 | 58 | 3.04 |  |
|  |  | Precentral Gyrus | L | -44 | 4 | 30 | 4.06 | 517 |
|  | Females (deactivation) | Angular Gyrus | R | 52 | -46 | 28 | 4.4 | 1684 |
|  |  | Middle Temporal Gyrus, temporooccipital part | R | 64 | -46 | 8 | 4.23 |  |
|  |  | Supramarginal Gyrus, posterior division | R | 66 | -40 | 38 | 3.41 |  |
|  |  | Cingulate Gyrus, posterior division | R | 8 | -36 | 46 | 4.28 | 1260 |
|  |  | (Temporal) Occipital Fusiform Cortex | R | 30 | -56 | -8 | 4.62 | 1259 |
|  |  | Middle Temporal Gyrus, temporooccipital part | L | -62 | -54 | 4 | 4.45 | 1222 |
|  |  | Angular Gyrus and Supramarginal Gyrus, posterior division | L | -52 | -52 | 22 | 3.93 |  |
|  |  | Superior Temporal Gyrus, posterior division | L | -66 | -32 | 0 | 3.24 |  |
|  |  | Temporal Occipital Fusiform Cortex | L | -26 | -54 | -10 | 4.71 | 1199 |
|  |  | Superior Temporal Gyrus, anterior division | R | 60 | -4 | -12 | 4.12 | 858 |
|  |  | Middle Temporal Gyrus, anterior division | R | 60 | -4 | -18 | 3.97 |  |
|  |  | Temporal Pole | R | 56 | 8 | -16 | 3.4 |  |
|  |  | Lateral Occipital Cortex, superior division | R | 34 | -76 | 22 | 3.62 | 794 |
|  |  | Occipital Pole | R | 16 | -90 | 10 | 3.59 |  |
|  |  | Cuneal Cortex | R | 14 | -82 | 28 | 3.3 |  |
|  |  | Superior Temporal Gyrus, anterior division | L | -58 | -2 | -8 | 3.81 | 479 |
|  | Males (deactivation) | (Temporal) Occipital Fusiform Gyrus | R | 24 | -74 | -10 | 4.2 | 1064 |
|  |  | Angular Gyrus | R | 52 | -44 | 24 | 3.67 | 701 |
|  |  | Supramarginal Gyrus, posterior division | R | 64 | -44 | 12 | 3.59 |  |
|  |  | (Temporal) Occipital Fusiform Gyrus | L | -24 | -78 | -12 | 4.02 | 525 |
|  |  | Lingual Gyrus | L | -22 | -48 | -10 | 3.52 |  |

**Table S4** Sex differences in brain (de)activation for the contrast Same Sex other body (80-100% morphed) > Own body (0% Morph)

| Presentation  Time | Group contrast | Region | Side | x | y | z | Z-max | Size (vox) |
| --- | --- | --- | --- | --- | --- | --- | --- | --- |
| 2s (Long) | Females > Males | No sig. |  |  |  |  |  |  |
|  | Males > Females | No sig. |  |  |  |  |  |  |
|  | Males (activation) | Temporal Occipital Fusiform Cortex, Inferior Temporal Gyrus, temporooccipital part | L | -44 | -56 | -14 | 3.77 | 989 |
|  |  | Lateral Occipital Cortex, inferior division | L | -48 | -70 | -4 | 3.55 |  |
|  |  | Occipital Fusiform Gyrus | L | -28 | -64 | -12 | 3 |  |
|  |  | Temporal Fusiform Cortex, posterior division | L | -36 | -40 | -20 | 2.9 |  |
|  |  | Temporal Occipital Fusiform Cortex, Temporal Fusiform Cortex, posterior division | R | 34 | -40 | -20 | 3.71 | 906 |
|  |  | Occipital Fusiform Gyrus | R | 26 | -76 | -12 | 3.32 |  |
|  |  | Precentral Gyrus | L | -52 | 8 | 30 | 3.33 | 717 |
|  |  | Inferior Frontal Gyrus, pars opercularis | L | -36 | 16 | 26 | 3.25 |  |
|  |  | Frontal Pole | L | -46 | 42 | 6 | 3.1 |  |
|  | Females (activation) | No sig. |  |  |  |  |  |  |
|  | Males (deactivation) | No sig. |  |  |  |  |  |  |
|  | Females (deactivation) | No sig. |  |  |  |  |  |  |
| 0.5s (Short) | Females > Males | Cerebellum | R | 38 | -70 | -38 | 3.67 | 3664 |
|  |  | Lingual Gyrus | L | -10 | -66 | 0 | 3.66 |  |
|  |  | Frontal Pole | R | 4 | 64 | 28 | 3.95 | 2662 |
|  |  | Precuneous Cortex | L | -4 | -72 | 54 | 3.31 | 1355 |
|  |  | Cingulate Gyrus, posterior division | L | -8 | -48 | 34 | 3.22 |  |
|  |  | Lateral Occipital Cortex, superior division | L | -46 | -68 | 40 | 3.27 | 991 |
|  |  | Middle Frontal Gyrus | L | -36 | 28 | 40 | 3.55 | 868 |
|  |  | Lateral Occipital Cortex, superior division | R | 48 | -66 | 42 | 3.56 | 653 |
|  |  | Frontal Pole | L | -42 | 54 | -8 | 3.31 | 508 |
|  | Males > Females | No sig. |  |  |  |  |  |  |
|  | Females (activation) | Middle Frontal Gyrus | L | -48 | 16 | 42 | 4.86 | 13215 |
|  |  | Frontal Pole | R | 22 | 40 | 48 | 4.77 |  |
|  |  | Cerebellum | L | -40 | -70 | -40 | 4.32 | 6800 |
|  |  | Precuneous Cortex | L | -8 | -56 | 32 | 4.33 | 3499 |
|  |  | Cingulate Gyrus, posterior division | R | 14 | -50 | 32 | 3.94 |  |
|  |  | Lateral Occipital Cortex, superior division | L | -44 | -62 | 40 | 4.5 | 2318 |
|  |  | Angular Gyrus | L | -48 | -52 | 54 | 3.89 |  |
|  |  | Lateral Occipital Cortex, superior division | R | 56 | -64 | 30 | 4.45 | 2210 |
|  |  | Frontal Pole | R | 48 | 50 | -8 | 3.54 | 1335 |
|  |  | Temporal Pole | R | 42 | 16 | -36 | 3.39 |  |
|  |  | Right Hippocampus, 1% Parahippocampal Gyrus, posterior division | R | 24 | -20 | -14 | 3.75 | 712 |
|  |  | Right Amygdala | R | 20 | -2 | -12 | 3.44 |  |
|  |  | Parahippocampal Gyrus, posterior division | R | 24 | -32 | -22 | 2.99 |  |
|  | Females (deactivation) | Postcentral Gyrus | L | -42 | -28 | 56 | 3.92 | 785 |
|  | Males (activation) | No sig. |  |  |  |  |  |  |
|  | Males (deactivation) | No sig. |  |  |  |  |  |  |

**Table S5** Sex differences in brain (de)activation for the contrast Opposite Sex other body (80-100%) > Scrambled image (control condition)

| Presentation  Time | Group contrast | Region | Side | x | y | z | Z-max | Size (vox) |
| --- | --- | --- | --- | --- | --- | --- | --- | --- |
| 2s (Long) | Males > Females | Lateral Occipital Cortex, superior division | L | -22 | -82 | 26 | 3.75 | 1396 |
|  |  | Cuneal Cortex | L | -14 | -78 | 26 | 3.45 |  |
|  |  | Precuneous Cortex | L | -6 | -78 | 52 | 3.42 |  |
|  |  | Precuneous Cortex | L | -2 | -38 | 48 | 4.13 | 1395 |
|  |  | Cingulate Gyrus, posterior division | R | 4 | -36 | 48 | 3.64 |  |
|  |  | Precentral Gyrus | L | -4 | -28 | 48 | 3.55 |  |
|  |  | Postcentral Gyrus | L | -4 | -40 | 66 | 3.35 |  |
|  |  | Middle Temporal Gyrus, temporooccipital part | L | -52 | -44 | 6 | 3.51 | 1020 |
|  |  | Superior Temporal Gyrus, posterior division | L | -48 | -40 | 2 | 3.44 |  |
|  |  | Postcentral Gyrus | R | 42 | -26 | 52 | 3.28 | 663 |
|  |  | Precentral Gyrus | R | 26 | -16 | 58 | 3.24 |  |
|  |  | Frontal Pole | L | -28 | 40 | 34 | 3.91 | 565 |
|  |  | Superior Frontal Gyrus | L | -14 | 34 | 40 | 2.98 |  |
|  |  | Angular Gyrus | R | 42 | -50 | 26 | 3.5 | 512 |
|  |  | Occipital Fusiform Gyrus | R | 18 | -80 | -10 | 3.21 | 488 |
|  |  | Occipital Pole | R | 6 | -96 | 16 | 3.1 |  |
|  |  | Lingual Gyrus | L | -14 | -86 | -4 | 3.05 |  |
|  |  | Intracalcarine Cortex | L | -10 | -86 | -2 | 2.97 |  |
|  | Females > Males | No sig. |  |  |  |  |  |  |
|  | Females (activation) | Lateral Occipital Cortex, inferior division | R | 40 | -82 | -6 | 5.81 | 3533 |
|  |  | Lateral Occipital Cortex, inferior division | L | -32 | -90 | -4 | 5.28 | 2493 |
|  |  | Lateral Occipital Cortex, superior division | R | 24 | -60 | 42 | 4.09 | 1082 |
|  |  | Vermis, Cerebellum |  | 0 | -56 | -32 | 3.39 | 689 |
|  |  | Cerebellum | L | -8 | -78 | -26 | 3.34 |  |
|  |  | Cerebellum | R | 8 | -50 | -30 | 2.98 |  |
|  |  | Precentral Gyrus | R | 40 | 8 | 28 | 4.93 | 508 |
|  |  | Middle Frontal Gyrus | R | 54 | 32 | 26 | 4.47 | 444 |
|  |  | Frontal Pole | R | 54 | 38 | 22 | 3.99 |  |
|  | Males (activation) | Lateral Occipital Cortex, inferior division | L | -44 | -78 | -12 | 5.5 | 7474 |
|  |  | Occipital Fusiform Gyrus | L | -40 | -70 | -12 | 5.24 |  |
|  |  | Occipital Pole | L | -36 | -92 | -8 | 4.74 |  |
|  |  | Temporal Occipital Fusiform Cortex | L | -40 | -46 | -18 | 4.6 |  |
|  |  | Lateral Occipital Cortex, inferior division | R | 50 | -76 | -10 | 5.72 | 4802 |
|  |  | Inferior Temporal Gyrus, temporooccipital part, and Temporal Occipital Fusiform Cortex | R | 44 | -60 | -12 | 5.58 |  |
|  |  | Postcentral Gyrus and Supramarginal Gyrus, anterior division | R | 56 | -20 | 42 | 4.45 | 2901 |
|  |  | Superior Parietal Lobule | R | 34 | -46 | 52 | 4.02 |  |
|  |  | Middle Frontal Gyrus | R | 46 | 30 | 22 | 4.64 | 2360 |
|  |  | Frontal Pole | R | 48 | 40 | 10 | 3.91 |  |
|  |  | Frontal Orbital Cortex | R | 24 | 32 | -16 | 3.45 |  |
|  |  | Precentral Gyrus | R | 50 | 8 | 24 | 3.36 |  |
|  |  | Right Thalamus | R | 10 | -16 | 10 | 3.97 | 1585 |
|  |  | Right Caudate | R | 14 | 18 | 8 | 3.77 |  |
|  |  | Left Caudate | L | -14 | 16 | 8 | 3.57 |  |
|  |  | Superior Frontal Gyrus and Paracingulate Gyrus | R | 4 | 52 | 24 | 3.75 | 1191 |
|  |  | Frontal Medial Cortex | R | 6 | 52 | -12 | 3.46 |  |
|  |  | Frontal Pole | L | -4 | 56 | -10 | 3.44 |  |
|  |  | Precentral Gyrus | L | -52 | 0 | 48 | 3.73 | 1022 |
|  |  | Inferior Frontal Gyrus, pars triangularis and Middle Frontal Gyrus | L | -50 | 26 | 24 | 3.52 |  |
|  |  | Postcentral Gyrus and Precentral Gyrus | R | 8 | -30 | 80 | 3.38 | 452 |
|  | Females (deactivation) | Occipital Pole | R | 14 | -92 | 12 | 5.28 | 16567 |
|  |  | Middle Temporal Gyrus, temporooccipital part and Supramarginal Gyrus, posterior division | R | 62 | -42 | 8 | 5.07 |  |
|  |  | Precuneous Cortex and Cingulate Gyrus, posterior division | R | 8 | -38 | 46 | 4.9 |  |
|  |  | Angular Gyrus | R | 62 | -50 | 20 | 4.88 |  |
|  |  | Middle Temporal Gyrus, temporooccipital part | L | -62 | -54 | 4 | 5.23 | 4528 |
|  |  | Angular Gyrus | L | -52 | -52 | 24 | 4.84 |  |
|  |  | Middle Frontal Gyrus | R | 32 | 34 | 36 | 4.59 | 1345 |
|  |  | Frontal Pole | R | 32 | 52 | 26 | 4.58 |  |
|  |  | Frontal Pole | L | -28 | 38 | 38 | 5.01 | 1151 |
|  |  | Middle Frontal Gyrus | L | -28 | 30 | 32 | 4.42 |  |
|  |  | Frontal Pole | L | -28 | 42 | 30 | 4.26 |  |
|  | Males (deactivation) | Middle Temporal Gyrus, temporooccipital part | R | 64 | -52 | 8 | 3.85 | 1173 |
|  |  | Angular Gyrus | R | 54 | -46 | 14 | 3.84 |  |
|  |  | Supramarginal Gyrus, anterior division | R | 66 | -26 | 24 | 3.78 |  |
| 0.5s (Short) | Females > Males | No sig. |  |  |  |  |  |  |
|  | Males > Females | No sig. |  |  |  |  |  |  |
|  | Females (activation) | Cerebellum | L | -6 | -76 | -24 | 3.73 | 920 |
|  |  | Lateral Occipital Cortex, inferior division | R | 54 | -62 | -2 | 3.58 | 668 |
|  |  | Frontal Pole | R | 44 | 38 | 16 | 4.02 | 535 |
|  | Males (activation) | Frontal Orbital Cortex | R | 24 | 30 | -10 | 3.41 | 813 |
|  |  | Right Caudate | R | 14 | 16 | 2 | 3.07 |  |
|  |  | Lateral Occipital Cortex, inferior division | L | -46 | -78 | 2 | 3.93 | 636 |
|  |  | Inferior Temporal Gyrus, temporooccipital part | L | -42 | -62 | -10 | 3.01 |  |
|  |  | Middle Temporal Gyrus, temporooccipital part | R | 50 | -58 | -2 | 3.86 | 592 |
|  |  | Lateral Occipital Cortex, inferior division | R | 44 | -64 | -8 | 3.3 |  |
|  |  | Inferior Temporal Gyrus, temporooccipital part | R | 46 | -56 | -10 | 3.07 |  |
|  | Females (deactivation) | Angular Gyrus and Supramarginal Gyrus | R | 52 | -46 | 30 | 4.37 | 1921 |
|  |  | Middle Temporal Gyrus, temporooccipital part | R | 64 | -42 | 4 | 3.93 |  |
|  |  | Superior Temporal Gyrus, anterior division | R | 56 | 0 | -14 | 3.81 |  |
|  |  | Middle Temporal Gyrus, temporooccipital part | L | -62 | -52 | 2 | 4.28 | 1815 |
|  |  | Angular Gyrus and Supramarginal Gyrus | L | -56 | -54 | 44 | 3.77 |  |
|  |  | Superior Temporal Gyrus, posterior division | L | -52 | -40 | 4 | 3.59 |  |
|  |  | (Temporal) Occipital Fusiform Cortex | R | 30 | -56 | -8 | 4.27 | 1462 |
|  |  | Temporal/Occipital Fusiform Cortex | L | -24 | -52 | -10 | 4.3 | 730 |
|  | Males (deactivation) | Temporal/Occipital Fusiform Gyrus | R | 28 | -62 | -10 | 3.98 | 1035 |

**Table S6** Sex differences in brain (de)activation for the contrast Opposite Sex other body (80-100% morphed) > Own body (0% Morph)

| Presentation  Time | Group contrast | Region | Side | x | y | z | Z-max | Size (vox) |
| --- | --- | --- | --- | --- | --- | --- | --- | --- |
| 2s (Long) | Males > Females | Cuneal Cortex and Precuneous Cortex | R | 4 | -78 | 36 | 3.49 | 1352 |
|  |  | Supracalcarine Cortex | R | 2 | -72 | 16 | 3.34 |  |
|  |  | Intracalcarine Cortex and Lingual Gyrus | R | 4 | -72 | 8 | 3.1 |  |
|  |  | Lateral Occipital Cortex, superior division | R | 24 | -84 | 22 | 3.09 |  |
|  | Females > Males | No sig. |  |  |  |  |  |  |
|  | Males (activation) | Supracalcarine Cortex |  | 0 | -70 | 18 | 3.84 | 3411 |
|  |  | Occipital Pole | R | 8 | -88 | 18 | 3.7 |  |
|  |  | Cuneal Cortex | L | -8 | -82 | 24 | 3.62 |  |
|  |  | Supramarginal Gyrus, posterior division and Angular Gyrus | R | 60 | -44 | 34 | 3.8 |  |
|  |  | Middle Temporal Gyrus, temporooccipital part | R | 62 | -46 | 8 | 3.75 | 2864 |
|  |  | Angular Gyrus | R | 58 | -52 | 18 | 3.63 |  |
|  |  | Middle Temporal Gyrus, anterior division | R | 54 | -4 | -24 | 3.59 |  |
|  |  | Middle Temporal Gyrus, posterior division | R | 60 | -20 | -8 | 3.58 |  |
|  |  | Angular Gyrus | L | -60 | -56 | 14 | 3.86 | 614 |
|  |  | Middle Temporal Gyrus, temporooccipital part | L | -62 | -56 | 10 | 3.82 |  |
|  |  | Supramarginal Gyrus, posterior division | L | -58 | -48 | 18 | 3.18 |  |
|  |  | Lateral Occipital Cortex, superior division | L | -56 | -62 | 22 | 2.86 |  |
|  | Females (activation) | No sig. |  |  |  |  |  |  |
|  | Females (deactivation) | Frontal Operculum Cortex and Insular Cortex | R | 36 | 22 | 4 | 5.02 | 15805 |
|  |  | Paracingulate Gyrus | R | 4 | 34 | 36 | 4.67 |  |
|  |  | Paracingulate Gyrus and Cingulate Gyrus, anterior division | R | 10 | 32 | 26 | 4.59 |  |
|  |  | Frontal Orbital Cortex | L | -32 | 22 | -10 | 4.57 |  |
|  |  | Cerebellum | L | -38 | -66 | -42 | 4.05 | 4015 |
|  |  | Lingual Gyrus and Occipital Fusiform Gyrus | R | 8 | -78 | -16 | 3.72 |  |
|  |  | Precentral Gyrus and Postcentral Gyrus | L | -32 | -26 | 56 | 4.24 | 2186 |
|  |  | Superior Parietal Lobule | L | -26 | -54 | 48 | 3.81 |  |
|  |  | Precuneous Cortex | L | -4 | -74 | 50 | 3.53 |  |
|  |  | Supramarginal Gyrus | R | 52 | -26 | 40 | 4.07 | 1499 |
|  |  | Superior Parietal Lobule | R | 28 | -54 | 52 | 3.87 |  |
|  |  | Lateral Occipital Cortex, superior division | R | 36 | -58 | 52 | 3.67 |  |
|  | Males (deactivation) | Paracingulate Gyrus |  | 0 | 22 | 40 | 3.25 | 712 |
|  |  | Cingulate Gyrus, anterior division | L | -8 | 24 | 30 | 3.23 |  |
|  |  | Frontal Pole | R | 50 | 36 | 18 | 3.97 | 691 |
|  |  | Middle Frontal Gyrus | R | 44 | 34 | 26 | 3.58 |  |
|  |  | Insular Cortex | R | 32 | 24 | -4 | 2.89 |  |
|  |  | Lateral Occipital Cortex, superior division | R | 32 | -64 | 46 | 3.46 | 438 |
|  |  | Superior Parietal Lobule | R | 28 | -52 | 46 | 3.45 |  |
| 0.5s (Short) | Females > Males | Precuneous Cortex | L | -4 | -72 | 54 | 3.91 | 1159 |
|  |  | Lateral Occipital Cortex, superior division | L | -10 | -68 | 62 | 3.31 |  |
|  | Males > Females | No sig. |  |  |  |  |  |  |
|  | Females (activation) | Cingulate Gyrus, posterior division | L | -12 | -54 | 30 | 4.21 | 5364 |
|  |  | Precuneous Cortex |  | 0 | -68 | 34 | 3.89 |  |
|  |  | Insular Cortex | R | 38 | -18 | 10 | 3.9 | 2149 |
|  |  | Parahippocampal Gyrus, posterior division | R | 22 | -36 | -16 | 3.48 |  |
|  |  | Frontal Pole |  | 0 | 54 | -6 | 4.25 | 1848 |
|  |  | Paracingulate Gyrus | R | 10 | 54 | 8 | 3.46 |  |
|  |  | Lateral Occipital Cortex, superior division | L | -42 | -66 | 24 | 4.14 | 1359 |
|  |  | Angular Gyrus | L | -46 | -56 | 32 | 3.56 |  |
|  |  | Lateral Occipital Cortex, superior division | R | 48 | -62 | 22 | 4.62 | 1222 |
|  |  | Lingual Gyrus | L | -32 | -42 | -6 | 3.32 | 1109 |
|  |  | Parahippocampal Gyrus, anterior division | L | -28 | -12 | -28 | 3.2 |  |
|  | Males (activation) | Frontal Pole | R | 8 | 58 | 28 | 3.28 | 587 |
|  |  | Superior Frontal Gyrus | R | 4 | 48 | 40 | 3.03 |  |
|  | Females (deactivation) | Postcentral Gyrus | L | -42 | -26 | 56 | 4.57 | 1328 |
|  |  | Postcentral Gyrus | L | -44 | -26 | 66 | 4.53 |  |
|  |  | Superior Frontal Gyrus | L | -20 | -6 | 58 | 3.11 |  |
|  |  | Precentral Gyrus | L | -40 | -10 | 66 | 3.01 |  |
|  |  | Superior Frontal Gyrus | R | 6 | 24 | 50 | 3.69 | 959 |
|  |  | Paracingulate Gyrus | R | 4 | 30 | 40 | 3.4 |  |
|  | Males (deactivation) | Precuneous Cortex | L | -4 | -72 | 54 | 4.07 | 1537 |
|  |  | Lateral Occipital Cortex, superior division | L | -16 | -68 | 58 | 3.58 |  |
|  |  | Paracingulate Gyrus | R | 6 | 18 | 44 | 4.25 | 1348 |
|  |  | Frontal Pole | R | 34 | 58 | 14 | 3.56 | 582 |
|  |  | Precentral Gyrus and Middle Frontal Gyrus | L | -30 | -6 | 58 | 3.58 | 502 |
|  |  | Superior Frontal Gyrus and Middle Frontal Gyrus | L | -26 | 2 | 58 | 3.29 |  |

**Table S7** Brain activation for the contrast Same Sex other body (any morph degree) parametrically-modelled ratings

| Presentation  Time | Group contrast | Region | Side | x | y | z | Z-max | Size (vox) |
| --- | --- | --- | --- | --- | --- | --- | --- | --- |
| 2s (Long) | Females (Correlation to "Me" Rating) | Postcentral Gyrus | L | -40 | -30 | 68 | 3.51 | 546 |
|  | Males (Correlation to "Me" Rating) | Postcentral Gyrus and Precentral Gyrus | L | -34 | -26 | 54 | 4.59 | 1972 |
|  | Females (Correlation to "Not Me" Rating) | Precuneous Cortex | R | 10 | -60 | 52 | 4.52 | 1373 |
|  |  | Middle Frontal Gyrus | L | -34 | 6 | 56 | 3.53 | 526 |
|  |  | Middle Frontal Gyrus | R | 36 | 10 | 44 | 3.25 | 523 |
| 0.5s (Short) | None Sig. |  |  |  |  |  |  |  |

**Table S8** Brain activation for the contrast Opposite Sex other body (any morph degree) parametrically-modelled ratings

| Presentation  Time | Group contrast | Region | Side | x | y | z | Z-max | Size (vox) |
| --- | --- | --- | --- | --- | --- | --- | --- | --- |
| 2s (Long) | Males (correlation to "not me" rating) > Females (correlation to "not me" ratings) | Cerebellum (but very dorsal, close to PCC/precuneus) | R | 4 | -52 | -10 | 3.78 | 7562 |
|  |  | Precuneous Cortex and Cingulate Gyrus, posterior division | R | 8 | -50 | 16 | 3.74 |  |
|  |  | Left Amygdala | L | -20 | -6 | -14 | 3.66 |  |
|  |  | Right Amygdala | R | 18 | 0 | -16 | 3.47 |  |
|  | Females (correlation to "me" rating) | Insular Cortex | R | 42 | 16 | -8 | 4.64 | 13939 |
|  |  | Left Caudate | L | -10 | 8 | -2 | 4.59 |  |
|  |  | Left Pallidum | L | -10 | 4 | -2 | 4.5 |  |
|  |  | Frontal Orbital Cortex | R | 28 | 20 | -14 | 4.38 |  |
|  |  | Brain-Stem | R | 2 | -20 | -22 | 4.36 |  |
|  |  | Insular Cortex | L | -30 | 20 | -4 | 4.26 |  |
|  |  | Cerebellum | L | -26 | -72 | -34 | 4.1 | 4660 |
|  |  | Cerebellum | R | 18 | -48 | -26 | 4.04 |  |
|  |  | Paracingulate Gyrus | L | -2 | 24 | 36 | 4.62 | 3536 |
|  |  | Cingulate Gyrus, anterior division | L | -6 | 30 | 26 | 4.45 |  |
|  |  | Superior Parietal Lobule | L | -30 | -56 | 44 | 4.54 | 3052 |
|  |  | Lateral Occipital Cortex, superior division | L | -26 | -74 | 50 | 4.3 |  |
|  |  | Supramarginal Gyrus, posterior division | L | -38 | -50 | 44 | 4.14 |  |
|  |  | Postcentral Gyrus | L | -44 | -28 | 58 | 4.06 |  |
|  |  | Supramarginal Gyrus, posterior division | R | 46 | -38 | 44 | 4.57 | 1561 |
|  |  | Lateral Occipital Cortex, superior division | R | 30 | -64 | 42 | 4.49 |  |
|  |  | Lateral Occipital Cortex, inferior division | R | 34 | -90 | -16 | 3.26 | 1026 |
|  |  | Inferior Temporal Gyrus, temporooccipital part | R | 58 | -56 | -24 | 2.99 |  |
|  |  | Occipital Pole | R | 22 | -96 | -12 | 2.97 |  |
|  | Males (correlation to "me" rating) | Paracingulate Gyrus and Cingulate Gyrus, anterior division | R | 4 | 18 | 38 | 4.42 | 1418 |
|  |  | Cingulate Gyrus, anterior division | R | 6 | 28 | 22 | 4.15 |  |
|  |  | Frontal Pole | R | 46 | 42 | 20 | 3.95 | 757 |
|  |  | Middle Frontal Gyrus | R | 44 | 34 | 20 | 3.64 |  |
|  |  | Insular Cortex | L | -28 | 24 | 0 | 3.76 | 655 |
|  |  | Frontal Orbital Cortex | L | -36 | 24 | -4 | 3.75 |  |
|  |  | Frontal Operculum Cortex | L | -34 | 26 | 4 | 3.7 |  |
|  |  | Frontal Orbital Cortex | R | 36 | 30 | -4 | 3.74 | 628 |
|  |  | Frontal Operculum Cortex | R | 46 | 16 | 2 | 3.42 |  |
|  |  | Insular Cortex | R | 38 | 16 | -4 | 3.09 |  |
|  | Females (correlation to "not me" rating) | Superior Parietal Lobule and Supramarginal Gyrus, posterior division | R | 24 | -24 | 64 | 3.61 | 2118 |
|  |  | Superior Parietal Lobule | L | -12 | -44 | 32 | 3.53 |  |
|  |  | Lateral Occipital Cortex, superior division | R | 4 | -22 | 54 | 3.48 |  |
|  |  | Occipital Pole | R | 30 | -32 | 58 | 3.32 |  |
|  |  | Lateral Occipital Cortex, superior division | L | -40 | -66 | 20 | 3.92 | 1947 |
|  |  | Angular Gyrus | L | -62 | -58 | 2 | 3.67 |  |
|  |  | Supramarginal Gyrus, posterior division | L | -62 | -48 | 34 | 3.49 |  |
|  |  | Supramarginal Gyrus, posterior division and Middle Temporal Gyrus, temporooccipital part | R | 48 | -60 | 12 | 4.11 | 1793 |
|  |  | Angular Gyrus | R | 36 | -20 | 14 | 3.73 |  |
|  |  | Middle Temporal Gyrus, temporooccipital part | L | -18 | -88 | 26 | 3.53 | 500 |
|  |  | Angular Gyrus and Supramarginal Gyrus, posterior division | L | -12 | -88 | 32 | 3.52 |  |
|  |  | Precuneous Cortex | L | -8 | -86 | 26 | 3.26 |  |
|  |  | Cingulate Gyrus, posterior division | L | -18 | -94 | 18 | 3.18 |  |
|  | Males (correlation to "not me" rating) | Middle Temporal Gyrus, temporooccipital part | R | 60 | -58 | 12 | 4.51 | 9739 |
|  |  | Lateral Occipital Cortex, superior division | R | 58 | -60 | 22 | 4.14 |  |
|  |  | Angular Gyrus | R | 52 | -58 | 20 | 4.09 |  |
|  |  | Cingulate Gyrus, posterior division | R | 6 | -50 | 22 | 3.79 |  |
|  |  | Precuneous Cortex | R | 6 | -52 | 6 | 3.75 |  |
|  |  | Middle Temporal Gyrus, anterior division | L | -60 | -6 | -18 | 3.67 |  |
|  |  | Lateral Occipital Cortex, superior division | L | -50 | -64 | 20 | 3.62 | 1428 |
|  |  | Middle Temporal Gyrus | L | -52 | -52 | 0 | 3.48 |  |
|  |  | Frontal Medial Cortex | L | -2 | 54 | -12 | 4.02 | 1226 |
|  |  | Frontal Pole | L | -2 | 58 | -14 | 3.85 |  |
|  |  | Paracingulate Gyrus | R | 2 | 54 | -2 | 3.45 |  |
|  |  | Precentral Gyrus | R | 24 | -26 | 54 | 3.78 | 881 |
|  |  | Superior Parietal Lobule | R | 28 | -46 | 70 | 3.51 |  |
|  |  | Postcentral Gyrus | R | 34 | -38 | 60 | 3.43 |  |
| 0.5s (Short) | Males (correlation to "me" rating) | Superior Frontal Gyrus | R | 22 | 14 | 62 | 3.48 | 541 |
|  |  | Middle Frontal Gyrus | R | 36 | 6 | 48 | 2.92 |  |

**Table S9** Brain activation for the contrast Own body (0% Morph) > Scrambled image, with age included as covariate of no interest.

| Group | Z-cluster threshold | Region | Side | x | y | z | Z-max | Cluster size† |
| --- | --- | --- | --- | --- | --- | --- | --- | --- |
| Females (Activation) | 2.3 | Insular cortex | R | 36 | 22 | -6 | 6.73 | 39818 |
|  |  | Superior Parietal Lobule, Angular Gyrus | R | 36 | -48 | 42 | 5.1 | 3395 |
|  |  | Paracingulate gyrus, anterior cingulate gyrus | M | 0 | 22 | 40 | 5.08 | 2857 |
| Males (Activation) | 2.3 | Lateral Occipital Cortex, inferior division | R | 40 | -84 | -6 | 6.42 | 32227 |
|  |  | Paracingulate gyrus, anterior cingulate gyrus | R | 6 | 24 | 38 | 5.05 | 1731 |
| Females (Deactivation) | 2.3 | Middle temporal gyrus | R | 68 | -44 | 4 | 5.66 | 4669 |
|  |  | Middle temporal gyrus | L | -64 | -56 | 6 | 5.4 | 3830 |
|  |  | Occipital Pole | L | -8 | -94 | 14 | 5.42 | 3487 |
|  |  | Precuneus | M | 0 | -54 | 54 | 5.76 | 2455 |
|  |  | Temporal Occipital Fusiform cortex (FBA) | R | 32 | -48 | -12 | 5.68 | 823 |
| Males (Deactivation) | 2.3 | Middle temporal gyrus | R | 68 | -44 | 4 | 5.39 | 3651 |
|  |  | Middle temporal gyrus | L | -64 | -56 | 6 | 5.09 | 1650 |
|  |  | Occipital Pole | R | 12 | -90 | 12 | 5.74 | 1493 |
|  |  | Temporal Occipital Fusiform cortex (FBA) | L | -28 | -48 | -8 | 4.7 | 819 |
|  |  | Temporal Occipital Fusiform cortex (FBA) | R | 28 | -54 | -14 | 5.07 | 788 |

**Table S10** Sex differences in brain activation for contrasts of the Body Perception Task, including age as covariate of no interest.

| Contrast | Group | Z-cluster threshold | Region | Side | x | y | z | Z-max | Cluster size† |
| --- | --- | --- | --- | --- | --- | --- | --- | --- | --- |
| Same sex Morph > Scrambled | M > F | 2.3 | Lateral Occipital Cortex, superior division | L | -36 | -84 | 36 | 4.43 | 1095 |
|  |  |  | Posterior cingulate gyrus /precuneus | L | -2 | -42 | 38 | 4.72 | 621 |
| Same sex Morph > Own body | M > F | 2.3 | Lateral Occipital Cortex (EBA) | L | -42 | -80 | -8 | 4.02 | 1226 |
|  |  |  | Temporal Occipital Fusiform Cortex (FBA) | R | 40 | -42 | -20 | 3.79 | 487 |
| Opposite sex Morph > Scrambled | M > F | 2.3 | Posterior cingulate gyrus /precuneus | L | -2 | -42 | 46 | 4.7 | 15162 |
|  |  |  | Postcentral gyrus/precentral gyrus | R | 40 | -26 | 60 | 4.38 | 2043 |
|  |  |  | Frontal pole | L | -28 | 62 | -6 | 3.92 | 1986 |
|  |  |  | Inferior frontal gyrus | L | -40 | 10 | 20 | 4.32 | 1173 |
|  |  |  | Middle frontal gyrus | L | -30 | 38 | 30 | 4.46 | 848 |
| Opposite sex Morph > Own body | M > F | 2.3 | Lingual gyrus | R | 4 | -74 | -2 | 4.25 | 3642 |
|  |  |  | Middle temporal gyrus, anterior division | R | 54 | 0 | -26 | 3.6 | 855 |
|  |  |  | Supramarginal gyrus, posterior division | R | 52 | -42 | 10 | 3.65 | 840 |
|  |  |  | Orbito-frontal cortex | L | -28 | 26 | -22 | 3.98 | 639 |
|  |  |  | Caudate nucleus | R | 16 | 20 | 0 | 3.47 | 588 |
|  |  |  | Frontal pole | R | 26 | 48 | 24 | 3.44 | 485 |
